# Immediate Psychological Responses to Aerobic and Resistance Exercise in People With Psychotic Disorders: A Randomized Controlled Trial in Psychiatric Rehabilitation

**DOI:** 10.1093/schbul/sbag012

**Published:** 2026-03-21

**Authors:** Nicole Korman, Justin Chapman, Ahmed Jerome Romain, Urska Arnautovska, Brendon Stubbs, Simon Rosenbaum, Dan J Siskind, Robert Stanton, Mike Trott

**Affiliations:** Metro South Addiction and Mental Health Service, Metro South Health, Brisbane, 4102, Australia; Queensland Centre for Mental Health Research, Wacol, 4076, Australia; Faculty of Medicine, Health and Behavioural Sciences, The University of Queensland, Brisbane, 4072, Australia; Metro South Addiction and Mental Health Service, Metro South Health, Brisbane, 4102, Australia; School of Pharmacy and Medical Sciences, Centre for Mental Health, Griffith University, Brisbane, QLD, 4111, Australia; Centre de recherche de l'Institut universitaire en santé mentale de Montréal, Montréal, Québec, H1N3V2, Canada; School of Kinesiology and Physical Activity Sciences, Université de Montréal, Montréal, Québec, H3T1J4, Canada; Metro South Addiction and Mental Health Service, Metro South Health, Brisbane, 4102, Australia; Queensland Centre for Mental Health Research, Wacol, 4076, Australia; Faculty of Medicine, Health and Behavioural Sciences, The University of Queensland, Brisbane, 4072, Australia; Department of Psychiatry, Psychology and Neuroscience, King’s College London, London, United Kingdom, SE58AF; Division of Social Psychiatry, Department of Psychiatry and Psychotherapy, Medical University of Vienna, Vienna, 1090, Austria; Comprehensive Center for Clinical Neurosciences and Mental Health, Medical University of Vienna, Vienna, 1090, Austria; Nutrition, Exercise and Social Equity Research Group, Discipline of Psychiatry and Mental Health, School of Clinical Medicine, University of New South Wales, Sydney, 2052, Australia; Metro South Addiction and Mental Health Service, Metro South Health, Brisbane, 4102, Australia; Queensland Centre for Mental Health Research, Wacol, 4076, Australia; Faculty of Medicine, Health and Behavioural Sciences, The University of Queensland, Brisbane, 4072, Australia; School of Health, Medical, and Applied Sciences, Central Queensland University, Rockhampton, QLD, 4701, Australia; Cluster for Resilience and Wellbeing, Appleton Institute, Central Queensland University, Wayville, South Australia, 5034, Australia; Metro South Addiction and Mental Health Service, Metro South Health, Brisbane, 4102, Australia; Queensland Centre for Mental Health Research, Wacol, 4076, Australia; Faculty of Medicine, Health and Behavioural Sciences, The University of Queensland, Brisbane, 4072, Australia

**Keywords:** resistance training, aerobic exercise, psychotic disorders, schizophrenia, acute exercise, psychiatric, rehabilitation

## Abstract

**Background and Hypothesis:**

People with psychotic disorders have limited strategies to manage acute psychological distress and emotions. Exercise has physical and mental health benefits but immediate psychological effects in this population remain underexplored. This study compared immediate psychological responses to resistance training (RT) and aerobic exercise among individuals with psychotic disorders in psychiatric rehabilitation and examined whether clinical characteristics were associated with these responses.

**Study Design:**

Fifty-three participants were randomized to RT or aerobic exercise. Immediate psychological responses—psychological distress, positive wellbeing, and fatigue—were assessed using the Subjective Exercise Experiences Scale at 2 timepoints within an 8-week trial (week 3: *n* = 52; week 8: *n* = 48), pre- and 10 min post-exercise. Baseline assessments included clinical and motivational variables. Primary analyses used linear mixed models for repeated measures.

**Study Results:**

Both exercise types led to large increases in positive wellbeing (RT: Hedges’ g = 1.24; aerobic: g = 1.31) and reductions in psychological distress (RT: g = –0.91; aerobic: g = –1.05). A significant group-by-time interaction was observed for fatigue, which increased in RT (g = 0.43) and decreased in aerobic (g = –0.58). Higher fatigue was associated with greater controlled motivation (β = 1.72) and depressed mood (β = 0.80) at baseline.

**Conclusions:**

RT and aerobic exercise improved positive wellbeing and reduced psychological distress whereas fatigue differed by exercise type. Findings suggest exercise may be a promising intervention for modulating immediate psychological responses in people with psychotic disorders in psychiatric rehabilitation.

## Introduction

People living with psychotic disorders including schizophrenia and related psychoses have a complex presentation including auditory hallucinations, delusions, disorganization, diminished activation, and reduced emotional expressiveness.^1^^,^^2^ These disorders contribute to significant functional impairment across employment, social, and daily life domains, and are associated with a high physical and psychological burden of disease.^3^ While antipsychotic medications can reduce psychotic symptoms,^4^ many individuals continue to experience high levels of psychological distress and emotional reactivity to daily stressors, but have a limited range of effective self-management techniques.^5^ Psychological distress can further impair functioning and impact recovery.^6–8^

Exercise can improve mental health and functioning in this population.^9–12^ Dropout from exercise trials for people with schizophrenia has been reported to range from 0% to 45%, with interventions that incorporate supervision from qualified professionals and motivational strategies having lower dropout rates.^13^ Positive psychological responses to a single bout of exercise (commonly termed “acute exercise” in the exercise science literature^14^^,^^15^) have been shown to predict subsequent exercise engagement,^16^ and to be associated with greater exercise self-efficacy.^17^ Conversely, negative responses following a single bout of exercise—such as fatigue or psychological distress—which we refer to here as *“*immediate psychological responses,” may deter future engagement.^18^ This may be especially relevant in psychosis, where reward-processing deficits^19^ and motivational challenges are common,^20^ making maintenance of exercise challenging.^21^ Few studies have investigated responses to a single bout of exercise in people with psychotic disorders or how different exercise modalities impact responses in this group, which could inform the development of interventions and self-management techniques for regulating psychological distress. A single bout of exercise has demonstrated benefits in distress modulation and enhancement of positive emotions in both the general population^15^^,^^18^ and for people with a range of mental illnesses, including anxiety, depression, post-traumatic stress disorder, and substance use disorders.^16^^,^^22–27^

For participants with psychotic disorders, one study compared immediate psychological responses to single bouts of high intensity interval training between participants with schizophrenia or major depressive disorders, finding that both groups improved psychological distress and positive wellbeing, but with a larger, more sustained response in depressed participants.^28^ Another study found improvements in immediate psychological responses such as positive wellbeing and distress from both yoga and aerobic exercise in people with schizophrenia.^29^ Heterogenous designs, comparator groups, and the focus on aerobic exercise or yoga has limited firm conclusions. Although relatively underexplored, resistance training (RT) has gained traction as having both physical and mental health benefits,^30–33^ however, the immediate psychological effects of RT in people with psychotic disorders have not previously been investigated.

With an increasing recognition of the role of exercise as an evidence-based psychiatric rehabilitation intervention,^34^ psychiatric rehabilitation services offer an ideal context to explore immediate psychological responses to a single bout of exercise due to their focus on supporting consumers to expand their repertoire of illness self-management skills.^35–37^ While exercise studies in these settings have demonstrated feasibility and physical health benefits,^38–41^ immediate psychological effects have received relatively little attention.

This study aimed to examine immediate psychological responses, including positive wellbeing, psychological distress, and fatigue across 2 single bouts of moderate-intensity RT versus aerobic exercise in individuals with psychotic disorders engaged in psychiatric rehabilitation. Given the exploratory nature of the research, no a priori hypotheses were proposed.

In other populations, such as individuals with depression, individual background, clinical and exercise prescription factors have influenced immediate psychological responses to a single bout of exercise.^15^ However, the current evidence base in people with psychotic disorders remains limited. Therefore, in addition to quantifying changes in immediate psychological responses and comparing these between exercise modalities, identifying potential clinical, demographic, and exercise-related factors associated with these responses may help inform the development of optimized exercise prescriptions in future research.

Hence, a secondary aim was to explore whether participants’ clinical characteristics—including illness factors, motivation, mood, exercise preferences, and ratings of perceived exercise intensity—were associated with changes in immediate psychological responses to a single bout of exercise. These analyses were considered exploratory, aimed at hypothesis generation rather than confirmatory inference.

## Methods

This trial was reported using the Consolidated Standards of Reporting Trials (CONSORT) checklist (Appendix 1).

### Trial Design

This study analyzed data nested within an 8-week randomized controlled trial investigating the feasibility of RT versus aerobic exercise. Ethical approval was provided by the local Human Research Ethics Committee (HREC/20267647); the parent and current study protocol were prospectively registered (ACTRN12620001309976). Full methods and primary outcomes are published elsewhere.^42^ All participants provided written informed consent. Rolling recruitment occurred from December 2020 to December 2023.

### Participants

Participants were referred by clinicians from 3 residential psychiatric rehabilitation units in Brisbane, Australia. These units provide accommodation and 24-h multidisciplinary mental health support to residents with low imminent risk to self or others.^43^^,^^44^ Eligible participants were aged 18-64 years, diagnosed with a DSM-5 psychotic spectrum disorder (eg, schizophrenia, schizoaffective disorder), cleared for exercise by an accredited exercise physiologist or general practitioner, in line with established pre-exercise screening guidelines^45^ and provided informed consent. Exclusions included pregnancy, a comorbid eating disorder contraindicating exercise, as determined by the treating psychiatrist, substance use interfering with rehabilitation, or inability to provide informed consent or understand instructions.

### Procedures

#### Randomization

Participants were randomized in permeated blocks of 4 at a 1:1 ratio to RT or aerobic exercise using a computer-generated sequence. Allocation codes were prepared by an independent biostatistician and provided in sealed envelopes. Due to the nature of the trial, participants could not be blinded to their allocation.

#### Intervention

Participants started a 30-min moderate-intensity RT or aerobic interval training program 3 times per week, consistent with evidence supporting physical and mental health benefits in individuals with schizophrenia.^46^ The initial prescription was aimed at an intensity and duration that was achievable for people with psychotic disorders with low cardiorespiratory fitness, in accordance with recommendations.^47^ The training duration was gradually progressed to 40 min per session in week 4 to a maximum of 45-55 min in week 8, based on participant response.^42^ Exercise was delivered on alternating days for 8 weeks. Both aerobic and RT interventions were designed in accordance with established guidelines.^48^^,^^49^ Sessions were delivered by an accredited exercise physiologist experienced in mental health settings. Intensity was guided using the modified Borg Category Ratio 10 scale Rating for Perceived Exertion (RPE),^50^ for which an RPE of 3-4 is equivalent to moderate intensity exercise, and RPE > 4 is considered vigorous.^48^ RPE is considered an appropriate adjunct measure of intensity where direct laboratory testing is not considered feasible.^51^ The initial prescription was aimed at an intensity achievable for participants with psychotic disorders with low physical conditioning but progressed to moderate intensity by week 3. Exercise was maintained as moderate (RPE = 3-4), though the workloads were individualized and progressed as the participant adapted to the intervention stimulus.^47^ Strategies to enhance adherence included the provision of behavioral support strategies recommended when engaging people with psychotic disorders in exercise, playing music of participants’ choice to enhance pleasure and choice of whether they preferred to exercise in groups or individually. For comprehensive details of both interventions, see Appendices 2-4.

### Measures

**Figure 1:**
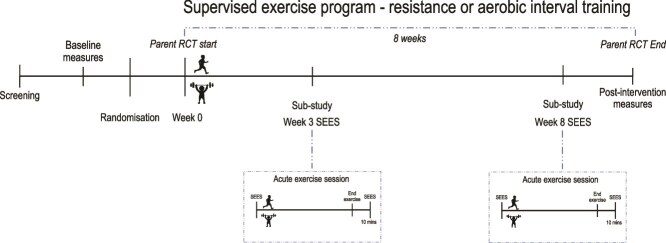
Relationship Between Parent and Substudy.

Participants completed the primary outcome measure (immediate psychological responses) of this study immediately before and 10-min after the first exercise session, which occurred in weeks 3 and 8 of the parent intervention. Week 3 was chosen as the first time point to allow participants time to familiarize themselves to the exercise type they had been in randomized to in the first 2 weeks of the parent study, and so that immediate psychological responses were to the exercise itself and not the learning process. Measures were administered to participants individually irrespective of group or individual exercise engagement, in the on-site gym or residential rehabilitation facilities. Participants received a $25 gift card at each timepoint as compensation for their time. Figure 1 shows the relationship between the current and parent study.

#### Primary Outcome Measure

*Subjective Exercise Experience Scale (SEES)* consists of 12 items that measure global psychological responses to exercise, with items assessing 3 domains—positive wellbeing, psychological distress, and fatigue, using a 7-point Likert scale (1 = not at all; 7 = very much so). The 3 subscales have been determined to have good internal consistency^52^^,^^53^ and have been used in previous research evaluating immediate responses to a single bout of exercise in the general populations^18^ and in people with schizophrenia.^28^^,^^29^

#### Baseline Measures

Assessments were collected prior to randomization at baseline, which was at week 0 of the 8-week exercise intervention (Figure 1). These included socio-demographics (eg, age, sex), clinical information (eg, diagnoses, medication use (converted to olanzapine equivalents),^54^ duration of illness, body mass index (BMI), and smoking status.

*Behavioral Regulation in Exercise Questionnaire* is a 24-item questionnaire used to assess behavioral regulations for exercise based on Self-Determination Theory that includes the following domains: amotivation, external, introjected, identified, integrated, and intrinsic behavioral regulations.^55^ Items are scored on a 5-point scale ranging from 0 (“Not true for me”) to 4 (“Very true to me”). Examples of questions include “I find exercise a pleasurable activity” and “I exercise because other people say I should.” These average domain scores were collapsed into the following 3 clinically relevant categories, as per previous research^56^^,^^57^: amotivation, controlled motivation (average of external and introjected) and autonomous motivation (average of identified, integrated, and intrinsic). BREQ-3 has high test–retest reliability (ρ > 0.7) and has been shown to be moderately predictive of exercise participation.^58^

*Brief Psychiatric Rating Scale* is an interviewer-rated scale for assessing change across a broad range of psychiatric symptoms, and has been used extensively across a variety of settings and patients.^59^ BPRS is rated used a structured interview guide and is scored out of 126, with higher scores indicating greater symptom severity, and ≥41 indicates at least moderate severity of illness. Item number 9 “depressed mood” (which assess sorrow, sadness, despondency, and pessimism out of a total score of 7) was used to assess mood at baseline, with higher ratings indicating greater depression.

*Scale for the Assessment of Negative symptoms* is a clinician-rated scale, designed to assess negative symptoms of schizophrenia widely used in schizophrenia research and across diverse populations.^60^ A higher score indicates greater severity of negative symptoms, with scores >63 indicative of “moderately ill.”^61^

*World Health Organization Disability Adjusted Scale (WHODAS) 2.0*: a 36-item interviewer-administered questionnaire to assess the overall health and disability in adult populations across different cultures. The WHODAS covers 6 domains of functioning including cognition, mobility, self-care, getting along, life activities, and participation.^62^

*Simple Physical Activity Questionnaire (SIMPAQ)*: a self-report physical activity questionnaire assessing the total time engaging in different physical activity domains and sedentary behavior in the previous week. Moderate-to-vigorous physical activity (MVPA) is calculated by summing walking and moderate to vigorous activity. The SIMPAQ has been internationally validated and is correlated with objective physical activity in people with severe mental illness.^63^

### Analysis

Statistical analysis was conducted using SPSS Statistics 24 and R Studio version 2025.05.1. Continuous variables were described using means and SDs, with normality tested using the Shapiro-Wilks test and Q-Q plot inspection. Demographic and clinical variables at baseline, and RPE at week 3 and week 8 were compared between groups using independent *t*-tests or Mann–Whitney U tests; categorical data was compared using Chi-square analysis.

The primary analyses consisted of 3 mixed models for repeated measures (MMRM), with one model per SEES domain (positive wellbeing, psychological distress, and fatigue). Each model included fixed effects for time, week, and intervention group. Age, sex, BMI, olanzapine equivalency were entered a priori as covariates due to their potential impact on psychological response to exercise.^64–66^ To identify additional covariates, univariate associations were conducted between baseline variables and changes in SEES domains using Spearman’s rank-order correlations (for continuous variables) and Kruskal–Wallis tests for categorical variables. Variables significantly associated with any SEES outcome were subsequently included as covariates in all 3 main models, alongside a time × week × intervention group interaction term (to examine whether pre/post changes differed between groups at week 3 and week 8). These analyses identified that baseline mood, duration of illness, controlled regulation, and self-reported MVPA were significantly associated with positive wellbeing, whereas amotivation was associated with psychological distress (See Appendix 6). A random effect for within-participant variation was included to account for repeated measures, and an unstructured covariance matrix was applied. Multicollinearity diagnostics indicated no evidence of collinearity among covariates, with all adjusted Generalized Variance Inflation Factors (GVIF^1/(2·Df)^) below 1.7, well under the conventional threshold of concern,^67^ (See Appendix 10). Estimated marginal means were derived from the MMRM models using the Kenward–Roger method for standard error estimation.^68^ Hedges *g* was calculated for pre/post change in each SEES domain for each group, with values of 0.2, 0.5, and 0.8 interpreted as small, medium, and large effect sizes, respectively.^69^ Due to this secondary analysis being exploratory, we did not correct for multiple comparisons.^70^ Univariate models (See Appendix 11) yielded results consistent with the multivariate analyses.

## Results

### Participants

**Figure 2:**
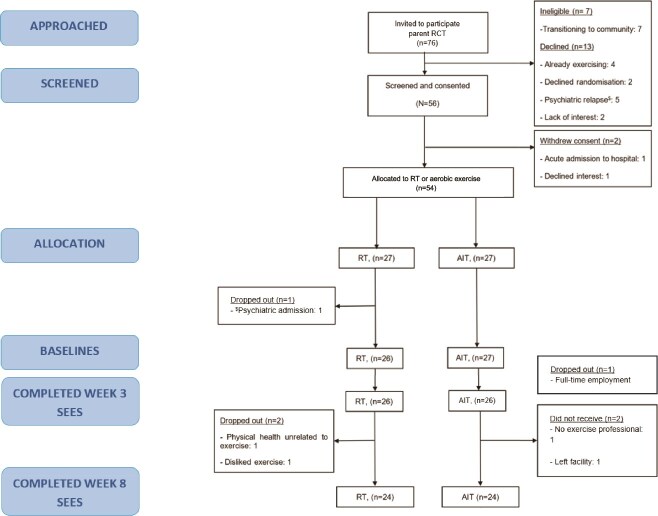
Consort Diagram.

Of 76 invited, 54 consented and 53 completed baseline measures. Six participants (12%) withdrew—3 from each condition. In RT, one withdrew due to dislike of the exercise and another for health reasons. Withdrawals from the aerobic condition were unrelated to health or exercise, occurring due to employment, facility discharge, or lack of staff during COVID-19.^42^ See Figure 2 for flowchart. Eight (15%) participants completed exercise in groups of 2 (2 groups of 2 in each exercise condition). Lockdown restrictions due to COVID 19 precluded groups for 2 years of the trial. The analysis included participants who completed the SEES at both time points—week 3 (*n* = 52) and week 8 (*n* = 48).

Median age of participants was 31 years (IQR 12), with 72% male, and 75% were diagnosed with schizophrenia or schizoaffective disorder (See Table 1 for baseline demographics). Median duration of illness was 5 years. Just under half (43.4%) were receiving clozapine, indicative of a treatment resistant illness.^71^ The majority (92.5%) had a baseline total symptom score of moderately unwell or worse, with a mean total symptom severity score consistent with “moderately-severe” threshold scores on the BPRS (≥53).^72^ Median depression scores were 2 (IQR = 3) categorized as “very mild” depressed mood.^73^ There were no differences between the groups other than for illness duration where RT participants had a longer duration of illness (Md (median) = 8.5 years, IQR (interquartile range) = 17) than the aerobic participants (Md = 3 years, IQR 6.5). Preference data revealed that 25% were randomly allocated their preference, 32% did not get allocated their preference and 43% had no preference for exercise type. Median RPE scores for both exercise groups were in the moderate intensity range (from 3 to 3.9), with no differences between exercise groups at either week 3 or week 8 (see Appendix 5).

**Table 1:** Baseline Clinical and Demographic Characteristics.

**Clinical and demographics**	**Total, *n* = 53**	**RT, *n* = 26**	**AIT, *n* = 27**
Age, years (IQR)^b^	31 (12)	33.5 (12)	28 (11)
Sex, male, *n* (%)	38 (71.6%)	21 (80.8%)	17 (63%)
**Clinical characteristics**			
Diagnosis: *n* (%)			
Schizophrenia	32 (60.3%)	19 (73.1%)	13 (48%)
Schizoaffective disorder	8 (15%)	3 (11.5%)	5 (18.5%)
BPAD with psychosis	5 (9.4%)	2 (7.7%)	3 (11.1%)
Major depression + psychosis	3 (5.6%)	1 (3.8%)	2 (7.4%)
Drug induced psychosis Psychotic disorder NOS	1 (1.8%)	0	1 (3.7%)
	2 (3.7%)	0	2 (7.4%)
First Episode psychosis	2 (3.7%)	1 (3.8%)	1 (3.7%)
Substance use disorder, yes, *n* (%)	5 (9.4%)	2 (7.7%)	3 (11.1%)
Duration illness, years (IQR)^a,b^	5 (11.5)	8.5 (17)	3 (6.5)
Olanzapine/clozapine/both, *n* (%)	33 (62.2%)	17 (65.4%)	16 (59%)
Clozapine, yes, *n* (%)	23 (43.4%)	14 (53.8%)	9 (33.3)
Olanzapine equivalents, mg, mean (SD)	25 (28.8)	31.3 (20)	26.6 (18)
Smoking, yes, (%)	20 (37.7%)	11 (42.3%)	9 (33.3%)
BMI, mean (SD)	32.3 (7.7)	32.4 (7.7)	31.6(7.9)
Waist circumference, cm, mean (SD)	105.9 (18.7)	108.5 (17.7)	103.5 (19.7)
Self-report MPVA, min/week (IQR)^b^	60 (150)	75 (199)	60 (90)
Self-report total PA, min/week (IQR)^b^	825 (768)	829 (591)	831 (511)
**Mental health**			
Global Functioning (WHODAS), mean (SD)	37 (20.2)	37.2(18.6)	38.1(22)
Total symptoms, (BPRS), mean (SD)	55 (12)	55 (17)	50 (20)
Moderate-severely unwell: (BPRS ≥41), yes (%)	49 (92.5)	26 (53.1)	23 (46.9)
Depressed mood, (BPRS)^b^	2 (3)	1 (3)	2(2)
Moderate-severely depressed (≥4/7), yes, (%)	13 (24.5)	8 (61.5)	5(38.5)
Negative symptoms (SANS), mean (SD)	26 (15)	28.6 (15)	24.1(14)
Amotivation (IQR)^b^	0 (0.5)	0.25 (1)	0 (0.5)
Controlled regulation (IQR)^b^	1.6(1.3)	1.5 (1.5)	1.6 (1.2)
Autonomous motivation (IQR)^b^	2.5 (1.1)	2.5 (0.9)	2.5(1.3)

Abbreviations: BMI = Body mass Index; BPAD – Bipolar Affective Disorder; MVPA = moderate/vigorous physical activity; NOS = not otherwise specified; PA = physical activity, SANS = Scale Assessment of Negative Symptoms; WHODAS = World Health Organisation Disability Assessment Schedule.

^a^ *P*-value significant difference between groups. ^b^Non-parametric statistics: median and IQR- interquartile range presented.

**Figure 3:**
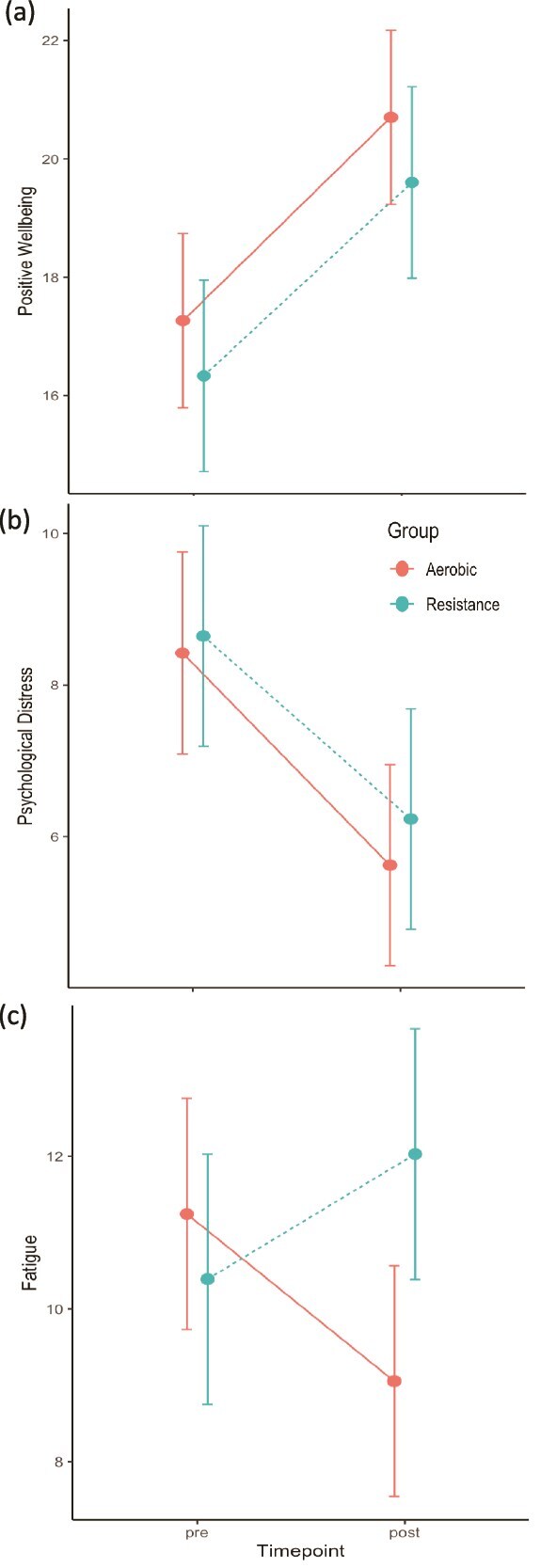
Pre versus Post (A) Positive Well-being Scores, (B) Psychological Distress Scores, and (c) Fatigue Scores Across Treatment Group^*^. ^*^Represents aggregated data from week 3 and week 8.

Self-report MVPA, mood, duration of illness, amotivation, and controlled motivation were identified as significant baseline covariates and were added to the model (see Appendix 6 for full matrix). There were no significant differences in changes in any of the 3 SEES domains between the exercise session for both exercise groups at week 3 or week 8, *P*-value ranges from .32 to .76 (see Appendix 7). Hence, we present combined results from week 3 and week 8 across each SEES domain for each exercise group (Table 2, Figure 3). Results demonstrating changes disaggregated by week 3 and week 8 can be found in the Appendices 8 and 9.

**Table 2:** Outcome Measures (LS Means).

	**Resistance training**			**Aerobic training**		
	**LSM pre (SE)**	**LSM post (SE)**	**Within-group** **Hedge’s g** **(95% CI)**	**LSM pre (SE)**	**LSM post (SE)**	**Within-group** **Hedge’s g** **(95% CI)**
Positive wellbeing	16.33 (0.81)	19.60 (0.81)	1.24 (0.85-1.64)	17.27 (0.73)	20.70 (0.73)	1.31 (0.91-1.70)
Psychological distress	8.64 (0.73)	6.23 (0.73)	−0.91 (−1.30 to −0.51)	8.42 (0.66)	5.62 (0.66)	−1.05 (−1.44 to −0.66)
Fatigue	10.39 (0.82)	12.03 (0.82)	0.43 (0.04-0.83)	11.25 (0.76)	9.06 (0.76)	−0.58 (−0.97 to −0.18)

Abbreviation: LSM = least squares mean. Within-group effect sizes calculated from LSMs and model-based residual SDs; between-group (interaction) effect sizes calculated from the model-based interaction estimate divided by the model-based residual SDs.

### Positive Wellbeing

There was a significant increase in positive wellbeing across both groups, (β = 2.92; SE = 0.73, *P* < .01), with large effect sizes for each group (RT Hedges g = 1.24 and aerobic Hedges g = 1.31, see Table 2). There were no differences between groups (*P* = .91) and no co-variates were significant (Appendix 7).

### Psychological Distress

There was a significant reduction in psychological distress across both groups (β = −2.41; SE = 0.41, *P* < .01), with large effect sizes for each group (RT Hedges g = −0.91 and aerobic Hedges g = −1.05, see Table 2). There were no differences between groups (*P* = .87) and no co-variates were significant (Appendix 7).

### Fatigue

Fatigue increased in RT (Hedges g = 0.43) but decreased in the aerobic group (Hedges g = - 0.58) following a single bout of exercise, with a significant between-group difference (β = 3.11; SE = 1.48, *P* = .04; between-group Hedges g = 0.43, See Table 2, Figure 3). Controlled motivation and depressed mood at baseline were significant co-variates for fatigue (*P* = .007 and .03, respectively).

## Discussion

This study compared the immediate psychological effects of moderate-intensity RT versus aerobic exercise in individuals with psychotic disorders in real world psychiatric rehabilitation settings. Both exercise types yielded significant increases in positive wellbeing and reductions in psychological distress, with no differences between modality or between exercise sessions at weeks 3 and 8, and with moderate to large effect sizes. However, differences were observed in the perception of fatigue which increased after RT and decreased after aerobic exercise.

### Positive Wellbeing and Psychological Distress

Improvements in positive wellbeing observed in this study align with the small number of previous investigations examining changes in positive responses to varied exercise bouts among people with psychotic disorders^28^^,^^29^^,^^74^ providing preliminary evidence that both RT and aerobic exercise may be beneficial. Similar large effects were seen in 2 of these studies investigating immediate psychological responses in people with psychotic disorders,^29^^,^^75^ with the remaining study reporting moderate effects.^28^ Although individuals with psychotic disorders can underestimate the potential positive experiences related to exercise because of alterations in reward prediction^20^ their experience of pleasure and positive emotions while performing the activity is comparable to the general population.^76^^,^^77^ Consistent with this, we observed large improvements in positive wellbeing and distress, similar to those found in healthy populations.^15^^,^^18^ Supportive interactions between the individual and the accredited exercise physiologist may have contributed to positive wellbeing improvements,^78^^,^^79^ which is an important aspect of implementing an exercise intervention in real-world clinical settings. Future studies could investigate unsupervised exercise to isolate the effect of exercise bouts or investigate methods for enhancing supportive interactions to maximize potential benefit.

Psychological distress decreased across both exercise groups, aligning with findings from yoga and aerobic exercise studies in people with psychotic disorders.^29^ General population studies report the potential for distress reduction from aerobic exercise,^18^ but with so few studies evaluating differences between exercise types in people with psychotic disorders, these novel findings remain preliminary. Despite most participants being moderately to severely unwell, and nearly half having treatment-resistant schizophrenia, improvements in psychological distress and positive wellbeing—alongside low dropout rates—underscore the acceptability of exercise as a means of modulating immediate psychological responses.

### Fatigue

There were differential perceptions of fatigue, which reduced post aerobic exercise and increased post RT. A previous review comprising mainly aerobic intervention supports the variable fatigue response to a single bout of exercise, with some studies reporting similar reductions in fatigue as we found. However more work is needed using RT interventions to confirm our findings.^18^ Despite individualized prescriptions, RT may still have been experienced as too intense for untrained participants because of low physical conditioning at baseline, with localized muscle fatigue (eg, biceps fatiguing from dumbbell curls) leading to the perception of greater overall fatigue.^80^ In the parent study^42^ RT was also associated with more delayed onset muscle soreness, which, while a temporary and expected response, may also have contributed to fatigue.^81^ It may also be that while fatigue occurs following a single bout, engagement in a longer-term exercise program may reduce perceptions of fatigue through familiarization and adaptation.^82^ Future studies should also address whether previous exercise experiences or familiarity with exercise modality influences immediate post session fatigue and whether fatigue is associated with longer-term adherence (ie, beyond 8 weeks).

We found that controlled motivation types (*I exercise because other people say I should/ I feel guilty when I don’t exercise*) was a significant covariate of fatigue. Previous research by our team found controlled motivation was positively associated with sedentary behavior and inversely associated with MVPA in a large international sample of people with mental illness.^56^ It may be that greater external motivation for exercise (including the use of authoritative or coercive techniques) can lead to negative psychological perceptions of exercise, such as fatigue, which may contribute to avoidance. While controlled motivation (eg, *because the doctor recommends it, or to reduce guilt*) has been positively associated with exercise uptake,^83^ strategies targeting autonomous motivation (i.e supporting exercise that is preferred, pleasurable, and for personally valued reasons) are more likely to successfully maintain exercise behavior^56^ and also lead to more favorable health outcomes,^84^ than prescriptive or coercive ones. The most autonomous form of motivation includes the experience of exercise that is enjoyable or pleasurable. This study found no association between autonomous motivation and changes in positive wellbeing, possibly due to the randomization process and its impact on autonomy or other aspects of the intervention, such as session duration or type of exercise.

We also found that depressed mood at baseline was associated with increases in fatigue following a single bout of exercise. Symptoms of depression commonly include feelings of low energy,^85^ hence those with greater ratings of depression may have been more prone to the psychological experience of fatigue immediately post exercise.

### Clinical Implications of Findings

The current findings highlight the potential utility of single bouts of exercise within psychiatric rehabilitation. Improvements in positive wellbeing and psychological distress following both aerobic and RT exercise suggest that bouts of these types of exercise may represent a possible non-pharmacological strategy to support emotional regulation in people with psychotic disorders. This has particular relevance given the widespread use of benzodiazepines to manage acute distress,^86^ which carry risks of dependence, sedation, and cognitive side-effects.^87^ There is also currently limited effectiveness of pharmacological treatments for negative symptoms.^88^ Behavioral activation therapy incorporates scheduled exercise bouts for the treatment of depressive disorders^89^ and our findings may suggest a potential role for single bouts of exercise to be used within behavioral activation frameworks to address negative symptoms of schizophrenia.^90^

At the same time, the observed increase in fatigue immediately following RT highlights the importance of tailoring exercise prescriptions. Since perceptions of fatigue can act as barriers to engagement,^91^ attempts to minimize negative psychological perceptions associated with exercise may require careful adjustments to intensity and/or session duration to optimize engagement of people with psychotic disorders.^20^ Moreover, recognizing that controlled forms of motivation and depressive symptoms were associated with greater fatigue, clinicians may need to prioritize strategies that enhance autonomous motivation and consider the impact of low mood when supporting exercise adoption.

Collectively, these findings suggest that exercise professionals and clinicians within psychiatric and rehabilitation settings could consider exercise as a non-pharmacological illness self-management option to modulate emotional responses, monitoring for perceptions of fatigue and modality specific responses. However, it would also be vital to explore the attitudes and preferences of people living with psychosis toward the single bouts of exercise for this purpose.

### Limitations and Future Directions

While this is the largest study to investigate immediate psychological responses to exercise in people with psychotic disorders to date, low statistical power was a notable limitation of the present study. This study is exploratory in nature, analyzing secondary SEES outcomes nested in a larger trial. Only 2 time points (weeks 3 and 8) were assessed. Although self-report scales such as the SEES capture important subjective experiences, their measurement sensitivity may be affected by response and social-desirability biases. Future studies could employ other intensive longitudinal measures such as ecological momentary assessment to improve sensitivity and ecological validity when capturing dynamic psychological responses.^17^ It was beyond the scope of this study to explore the biological or psychological mechanisms underpinning changes in psychological response from both aerobic and RT exercise, but this an important future line of enquiry, particularly the endocannabinoid system which has a pivotal role in reward learning.^92^

A significant strength of this study is the novelty of the included analysis. Immediate psychological responses to RT have not previously been evaluated in this cohort. However, the small sample size and multiple analyses highlight the need for replication with larger samples, and further exploration of factors such as session duration and intensity and comparison with other types of physical activities that may influence psychological responses, such as leisure and recreation. Immediate psychological responses may predict subsequent behavior and influence long-term exercise adherence through general principles of intrinsic reinforcement^93^ with the tendency for greater repetition of positive experiences and avoidance of negative ones. However, this study did not assess affective responses during exercise^94^ which has been found to be important for long-term adherence in the general population^95^ and should be considered in future research.^96^ We note participants were randomized to exercise groups, which limited autonomy. Future studies could explore immediate psychological responses when participants are able to choose their preferred exercise modality, as this may influence the magnitude of responses.

It was beyond the capacity of this study to investigate sustained psychological responses following RT or aerobic sessions (ie, greater than 10 min), however, this is clinically relevant to people with psychotic disorders and requires future investigation.

Our exercise sessions were scheduled as part of a structured trial rather than at a time of specifically identified distress. Future research should also investigate the use of different exercise types during periods of acute distress as identified by the individual, as this may offer further insights into the optimal way to implement single bouts of exercise as a therapeutic intervention within routine clinical settings. Exercise could also be compared with other conventional strategies for distress reduction, such as cognitive behavior therapy strategies (ie, hyperventilation control) or benzodiazepines to understand differential effects of interventions and hence inform optimal future psychiatric rehabilitation practices. As is commonly encountered in research in people with psychotic disorders, the sample was overrepresented by males, which should be taken into account when considering generalizability. While not statistically significant at baseline, future research should explore whether differences in sex, mood, or diagnosis influence immediate psychological responses. In this study the sample size was too small to perform subgroup analyses and these investigations were not planned a priori, however, this could be a future aim in larger samples. Similarly, the sample size was too small to explore whether exercising in a small group versus individually elicited differential effects; however, this represents an important avenue for future research to determine the optimal format for immediate psychological responses.

## Conclusions

This study found increases in positive wellbeing and reductions in psychological distress following single bouts of both RT and aerobic exercise, with no difference between exercise types, in a cohort of people with psychotic disorders accessing mental health rehabilitation services. RT resulted in greater perceptions of fatigue than aerobic exercise. Controlled motivation and depressed mood were associated with increases in perceived fatigue. These findings are preliminary and require replication. Future research should investigate immediate psychological responses to unsupervised exercise sessions and compare single bouts of aerobic and RT with other sources of psychological distress reduction.

## Supplementary Material

AppendicesSczBull_sbag012
