# Supplementary material for: Immediate Psychological Responses to Aerobic and Resistance Exercise in People With Psychotic Disorders: A Randomized Controlled Trial in Psychiatric Rehabilitation
Source: Schizophr Bull. 2026 Mar 21;52(2):sbag012. doi: 10.1093/schbul/sbag012 (PMC13005117; doi:10.1093/schbul/sbag012)
Supplement: AppendicesSczBull_sbag012 [file appendicessczbull_sbag012.docx]

List of Appendices

Table of Contents

[**Appendix 1.** CONSORT checklist. 2](#_Toc217230038)

[**Appendix 2**. ANZCTR Protocol 4](#_Toc217230039)

[**Appendix 3a.** Intervention structure. 33](#_Toc217230040)

[**Appendix 3b.** Training progression. 34](#_Toc217230041)

[**Appendix 4.** Proforma CERT checklist 35](#_Toc217230042)

[**Appendix 5**. Pre versus post (a) positive wellbeing scores, (b) psychological distress scores, and (c) fatigue scores across treatment group. 39](#_Toc217230043)

[**Appendix 6.** RPE data for week 3 and week 8. 40](#_Toc217230044)

[**Appendix 7**. Associations between baseline variables and dependent variables 41](#_Toc217230045)

[**Appendix 8.** Mixed Model Repeated Measures analysis 42](#_Toc217230046)

[**Appendix 9.** Table of outcome measures (LS means) week three and week eight. 43](#_Toc217230047)

[**Appendix 10.** Figures for week three and week eight – Pre versus post (a) positive wellbeing scores, (b) psychological distress scores, and (c) fatigue scores across treatment group. 44](#_Toc217230048)

[**Appendix 11.** Multicollinearity model. 45](#_Toc217230049)

[**Appendix 12.** Univariate analyses. 48](#_Toc217230050)

# **Appendix 1.** CONSORT checklist.


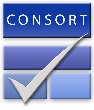
CONSORT 2010 checklist of information to include when reporting a randomised trial*

| Section/Topic | Item No | Checklist item | Reported on page No |
| --- | --- | --- | --- |
| Title and abstract | | | |
|  | 1a | Identification as a randomised trial in the title | 1. |
|  | 1b | Structured summary of trial design, methods, results, and conclusions (for specific guidance see CONSORT for abstracts) | 1. |
| Introduction | | | |
| Background and objectives | 2a | Scientific background and explanation of rationale | 2-3 |
|  | 2b | Specific objectives or hypotheses | 3 |
| Methods | | | |
| Trial design | 3a | Description of trial design (such as parallel, factorial) including allocation ratio | 5 |
|  | 3b | Important changes to methods after trial commencement (such as eligibility criteria), with reasons | N/A |
| Participants | 4a | Eligibility criteria for participants | 4 |
|  | 4b | Settings and locations where the data were collected | 5 |
| Interventions | 5 | The interventions for each group with sufficient details to allow replication, including how and when they were actually administered | 5 (and Appendix 2, 3, 4) |
| Outcomes | 6a | Completely defined pre-specified primary and secondary outcome measures, including how and when they were assessed | 5-6 |
|  | 6b | Any changes to trial outcomes after the trial commenced, with reasons | N/A |
| Sample size | 7a | How sample size was determined | 4 |
|  | 7b | When applicable, explanation of any interim analyses and stopping guidelines | N/A |
| Randomisation: |  |  |  |
| Sequence generation | 8a | Method used to generate the random allocation sequence | 5. |
|  | 8b | Type of randomisation; details of any restriction (such as blocking and block size) | 5. |
| Allocation concealment mechanism | 9 | Mechanism used to implement the random allocation sequence (such as sequentially numbered containers), describing any steps taken to conceal the sequence until interventions were assigned | 5 |
| Implementation | 10 | Who generated the random allocation sequence, who enrolled participants, and who assigned participants to interventions | 5. |
| Blinding | 11a | If done, who was blinded after assignment to interventions (for example, participants, care providers, those assessing outcomes) and how | 5. |
|  | 11b | If relevant, description of the similarity of interventions | 5, Appendix 3, |
| Statistical methods | 12a | Statistical methods used to compare groups for primary and secondary outcomes | 7-8 |
|  | 12b | Methods for additional analyses, such as subgroup analyses and adjusted analyses | N/A |
| Results | | | |
| Participant flow (a diagram is strongly recommended) | 13a | For each group, the numbers of participants who were randomly assigned, received intended treatment, and were analysed for the primary outcome | 8-9 Figure 1 |
|  | 13b | For each group, losses and exclusions after randomisation, together with reasons | 8-9, Figure 1 |
| Recruitment | 14a | Dates defining the periods of recruitment and follow-up | 4 |
|  | 14b | Why the trial ended or was stopped | N/A |
| Baseline data | 15 | A table showing baseline demographic and clinical characteristics for each group | 8,Table 1 |
| Numbers analysed | 16 | For each group, number of participants (denominator) included in each analysis and whether the analysis was by original assigned groups | Table 2 |
| Outcomes and estimation | 17a | For each primary and secondary outcome, results for each group, and the estimated effect size and its precision (such as 95% confidence interval) | Table 2, 9-10 |
|  | 17b | For binary outcomes, presentation of both absolute and relative effect sizes is recommended |  |
| Ancillary analyses | 18 | Results of any other analyses performed, including subgroup analyses and adjusted analyses, distinguishing pre-specified from exploratory |  |
| Harms | 19 | All important harms or unintended effects in each group (for specific guidance see CONSORT for harms) | 14 |
| Discussion | | | |
| Limitations | 20 | Trial limitations, addressing sources of potential bias, imprecision, and, if relevant, multiplicity of analyses | 13 |
| Generalisability | 21 | Generalisability (external validity, applicability) of the trial findings | 14. |
| Interpretation | 22 | Interpretation consistent with results, balancing benefits and harms, and considering other relevant evidence | 10-12 |
| Other information | | |  |
| Registration | 23 | Registration number and name of trial registry | 4 |
| Protocol | 24 | Where the full trial protocol can be accessed, if available | Appendix 2 |
| Funding | 25 | Sources of funding and other support (such as supply of drugs), role of funders | 13 |

*We strongly recommend reading this statement in conjunction with the CONSORT 2010 Explanation and Elaboration for important clarifications on all the items. If relevant, we also recommend reading CONSORT extensions for cluster randomised trials, non-inferiority and equivalence trials, non-pharmacological treatments, herbal interventions, and pragmatic trials. Additional extensions are forthcoming: for those and for up to date references relevant to this checklist, see [www.consort-statement.org](http://www.consort-statement.org).

# **Appendix 2**. ANZCTR Protocol

PROTOCOL TITLE

Resistance training versus aerobic exercise in a rehabilitation setting for people living with psychotic disorders: a randomised controlled trial

SHORT TITLE

Stand alone resistance training

Protocol ID: SART

Version: 2.0 Date: 7^th^ June 2023

Investigator Initiated Study

COODINATING PRINCIPAL INVESTIGATOR

Dr Nicole Korman

Consultant Psychiatrist

Metro South Addiction and Mental Health Service

Baragoola Street

Coorparoo QLD 4151

AUTHORS

Dr Nicole Korman

DOCUMENT HISTORY

| Version | Date | Summary of change |
| --- | --- | --- |
| 1.0 | 5^th^ August 2020 | Original |
| 1.1 | 18^th^ November 2020 | SEES and audio recording of interview |
| 2.0 | 7^th^ June 2023 | Change in research personnel and intervention |

Clinical Study Protocol Signature Page

Protocol ID: SART

Version: 18^th^ November 2020

coordinating Principal investigator:

dr Nicole korman

Metro South Addiction and Mental Health Service

I agree to:

Implement and conduct this study diligently and in strict compliance with the protocol, ICH Good Clinical Practice Guidelines, the Declaration of Helsinki and all applicable laws and regulations.

Maintain all information supplied by the Sponsor (or its delegate) in confidence and when this information is submitted to an approved Human Research Ethics Committee (HREC), it will be submitted with a designation that the material is confidential.

Coordinating Investigator Printed Name Signature Date

**Note** Confidential information contained herein is made available to you in your capacity as Investigator. It is provided only for review by you, your staff and approved Human Research Ethics Committee (HREC) members, or regulatory authorities. Except as necessary to obtain properly informed consent for participation, it is expected that there will be no disclosure to other persons.

INVESTIGATORS:

Coordinating Principal Investigator:

Dr Nicole Korman

Consultant Psychiatrist

Metro South Addiction and Mental Health Service

6 Baragoola Street Coorparoo QLD 4151

Phone: 0737277200

Email: [nicole.korman@health.qld.gov.au](http://www.consort-statement.org)

Associate Investigators:

Dr Justin Chapman

Consumer consultant, Metro South Addiction and Mental Health Services

Research Officer, QIMR

Phone: 07 31762111

Email: **justin.chapman@qimrbergohofer**.

Associate Professor Dan Siskind

Clinical Academic Psychiatrist

Metro South Addiction and Mental Health Service

Ipswich Road, Woollongabba, QLD 4151

Phone: 073176 2111

Email: [**dan.siskind@health.qld.gov.au**](mailto:dan.siskind@health.qld.gov.au)

Mr Steve Koh

Accredited Exercise Physiologist

Metro South Addiction and Mental Health Service

Bayside, Coorparoo and Logan Community Care Unit

Phone: 073176 2111

Email: stevenkoh10@outlook.com

Ms Cassandra Dodd

Accredited Exercise Physiologist

Metro South Addiction and Mental Health Service

Bayside, Coorparoo and Logan Community Care Unit

Phone: 073176 2111

Email: Cassandra.dodd@health.qld.gov.au

Ms Talia McIntosh

Accredited Exercise Physiologist

Metro South Addiction and Mental Health Service

Coorparoo Community Care Unit

Phone: 07 3727 7200

Email: talia.mcintosh2@health.qld.gov.au

Dr Frances Dark

Consultant Psychiatrist and Director

Metro South Addiction and Mental Health Service

Phone: 073176 2111

Email: **Frances.dark@health.qld.gov.au**

Dr Joe Firth

Presidential Fellow

University of Manchester

Email: **joefirth@gmail.com**

Dr Simon Rosenbaum

Associate Professor

University of New South Wales

Email: s.rosenbaum@unsw.edu.au

Table of Contents

[1 Introduction 8](#_Toc47699064)

[2 Objectives 10](#_Toc47699065)

[2.1 Primary Objectives 11](#_Toc47699066)

[2.2 Secondary Objectives 11](#_Toc47699067)

[3 Study Design 11](#_Toc47699068)

[4 Study Population 12](#_Toc47699069)

[4.1 Number of participants 12](#_Toc47699070)

[4.2 Inclusion Criteria 12](#_Toc47699071)

[4.3 Exclusion Criteria 12](#_Toc47699072)

[4.4 Screening assessment 13](#_Toc47699073)

[5 Participant Information and Informed Consent 14](#_Toc47699074)

[6 Study Assessments and Procedures 14](#_Toc47699075)

[6.1 Study Restrictions 18](#_Toc47699076)

[6.1.1 Adverse Events 18](#_Toc47699077)

[7 Investigational Exercise(s) 18](#_Toc47699078)

[7.1 Description of Investigational Exercise(s) 18](#_Toc47699079)

[7.1.1 Resistance training Intervention (RT) 19](#_Toc47699080)

[7.1.2 Moderate Intensity Aerobic Interval Training (MIIT) 20](#_Toc47699081)

[7.1.3 Fidelity 21](#_Toc47699082)

[7.1.4 Strategies to enhance adherence 21](#_Toc47699083)

[8 Adverse Events (AE) and Serious Adverse Events (SAE) 22](#_Toc47699084)

[8.1 Definition of an Adverse Event (AE) 22](#_Toc47699085)

[8.2 Definition of a Serious Adverse Event (SAE) 23](#_Toc47699086)

[8.3 Time Period, Frequency, and Method of Detecting AEs and SAEs 23](#_Toc47699087)

[8.4 Recording of AEs and SAEs 23](#_Toc47699088)

[8.5 Evaluating AEs and SAEs 24](#_Toc47699089)

[8.5.1 Assessment of Intensity 24](#_Toc47699090)

[8.5.2 Assessment of Causality 24](#_Toc47699091)

[8.6 Follow-up of AEs and SAEs 25](#_Toc47699092)

[8.7 Risk Management Process 25](#_Toc47699093)

[9 Participant Completion and Discontinuation 30](#_Toc47699094)

[9.1 Participant Completion 30](#_Toc47699095)

[9.2 Participant Withdrawal by the Investigator 30](#_Toc47699096)

[10 Case Report Form (CRF) 30](#_Toc47699097)

[11 Data Analysis and Statistical Considerations 30](#_Toc47699098)

[11.1 Hypotheses 30](#_Toc47699099)

[11.2 Endpoints 31](#_Toc47699100)

[11.2.1 Primary 31](#_Toc47699101)

[11.2.2 Secondary 31](#_Toc47699102)

[11.3 Sample Size and Power 31](#_Toc47699103)

[11.4 Statistical Analysis 31](#_Toc47699104)

[12 Data Management 32](#_Toc47699105)

[13 Monitoring and Quality Assurance 33](#_Toc47699106)

[14 Investigator Responsibility 33](#_Toc47699107)

[15 Study Report 33](#_Toc47699108)

[16 Administrative Procedures 34](#_Toc47699109)

[16.1 Ethical Considerations 34](#_Toc47699110)

[16.2 Ethical Review Committee 34](#_Toc47699111)

[16.3 Informed Consent 34](#_Toc47699112)

[16.4 Participant Reimbursement 35](#_Toc47699113)

[16.5 Investigator Indemnification 35](#_Toc47699114)

[16.6 Publication Policy 35](#_Toc47699115)

[16.7 Protocol Amendments 35](#_Toc47699116)

[16.8 Version Control 36](#_Toc47699117)

[16.9 Protocol Compliance 36](#_Toc47699118)

[16.10 Archives: Retention of Study Records 37](#_Toc47699119)

[17 References 38](#_Toc47699120)

**ABBREVIATIONS AND DEFINITIONS OF TERMS**

AEP – Accredited Exercise Physiologist

AIT – aerobic interval training

BPRS- Brief Psychiatric Rating Scale

PA – Physical activity

PI – Principal Investigator

RCT – Randomised Controlled Trial

RT- resistance training

SMI – Severe Mental Illness

SANS – Scale Assessment of Negative Symptoms

SIMPAQ – Simple Assessment of Physical Activity Questionnaire

Introduction

Chronic psychotic disorders, such as schizophrenia and related psychoses are complex and typically start early in adult life. Whilst considered a low prevalence disorder (1), the impact of schizophrenia through reduced quality of life, lost productivity and reliance on carers and the health service, can be very large (2). People with chronic psychotic disorders can experience significant burden from active symptoms of their mental illness, leading to functional impairment, and associated disability, with median social and clinical recovery considered to be as low as 13.5% (3).

Psychotropic medications and psychosocial therapies can successfully result in a reduction in many symptoms of psychotic disorders, such as hallucinations, delusions and depression, however recovery can be hampered by treatment resistance, incomplete response and distressing side effects of medications. Some specific symptom domains (such as negative and cognitive symptoms) are common (4), do not respond well to psychotropic medications and can be exacerbated by the medications themselves, with no currently accepted effective pharmacotherapy for negative or cognitive symptoms of schizophrenia (5, 6).

Low cardiorespiratory fitness and muscular weakness contribute to the very poor physical functioning and health of people with chronic psychotic disorders (7-9) which is in turn, associated with reduced global functioning (10). There is an urgent need to develop effective treatments that may augment existing first line treatments and improve functioning and recovery rates for people with chronic psychotic disorders.

In recent years, there has been increasing focus on the use of exercise interventions for people with chronic psychotic disorders, not only to improve the physical health of this population (11) but also as a strategy to improve mental health outcomes, with a growing evidence base regarding the positive impact of exercise on symptoms and functional outcomes (positive symptoms, negative symptoms, quality of life, functioning), (12, 13).

However, the vast majority of these interventions have investigated aerobic exercise protocols (13, 14) (defined as “exercise that uses oxygen to meet energy demands via aerobic metabolism to increase cardiorespiratory fitness”) or aerobic in combination with resistance training elements (resistance training defined as “repeated muscular movement that may increase muscle strength”), (15).

A 2015 meta-analysis found that exercise interventions above 90 mins of moderate to vigorous exercise per week was shown to improve positive, negative and mood symptoms in people with schizophrenia (13), although most of these trials involved continuous aerobic training. Scant attention has been paid to resistance training (RT) as a stand-alone treatment, identified as a significant gap in the literature and our understanding of the impact of this type of training in people with schizophrenia and psychotic disorders (16, 17).

In the general population hand grip strength (as a proxy measure of muscular strength) has been found to be inversely associated with mortality and may in fact be a better predictor of mortality than other conditions known to impact health such as hypertension or diabetes (18, 19). RT in the general population has resulted in improvements in both muscular performance and strength, with improvements also seen in chronic health conditions such as diabetes, cardiovascular disease, hypertension and metabolic abnormalities (20).

In clinical mental health populations such as people with depressive and anxiety disorders, there appears to be mounting evidence of improvements in mood, both in stand- alone RT and combination with aerobic interventions (21). In a large cross-sectional study involving people with schizophrenia, hand grip strength was found to be associated with cognitive outcomes such as working memory and processing speed (19). Preliminary evidence involving a small number of studies indicates the potential feasibility and safety of RT in this population (22,23), and that some people with schizophrenia may in fact express a preference for this type of training (24,25). A small number of trials have revealed the potential for physical health improvements such as power, strength and walking efficiency, which has promise given the potential impact of functional improvement on independent daily living tasks which can be challenging for people with chronic psychotic disorders (14, 22, 23, 26).

Further, only two randomised controlled trials have examined the impact of stand- alone RT on mental health outcomes, with conflicting results with respect to mental health symptoms, global functioning and quality of life (22, 23). Current recommendations for RT in people with psychotic disorders are derived from general population guidelines regarding physical health benefits of RT ie resistance training of main muscle groups at least two times per week. Whilst research regarding the impact of RT in depression and anxiety has revealed mental health benefits, few definitive conclusions can be drawn regarding about the impact of RT on mental health outcomes in people with psychotic disorders at this stage.

Another gap in the literature is that very few studies in people with chronic psychotic disorders have compared types of exercise with each other (27), with the majority of evidence regarding the benefits of exercise being derived from trials in which aerobic or combined aerobic and resistance exercise is compared with an inactive or non-exercise control. Specifically, few trials in people with schizophrenia have addressed comparisons between aerobic and resistance training protocols and those that have, have evaluated physical parameters such as brain volume or physical fitness, rather than mental health outcomes (28, 29).

Understanding the differential effects of resistance training versus aerobic exercise with respect to impact on functional and mental health outcomes is crucial to help guide optimal exercise prescription in this population and provide people with schizophrenia and related psychoses the best evidence regarding treatment options that may assist with recovery. Acknowledging the importance of enjoyment in long term adherence to exercise, of particular interest is whether this type of training can be enjoyed by people with psychotic disorders, as either complementary to, or as an alternative to more traditionally prescribed aerobic programs.

Community Care Units (CCU’s) are residential rehabilitation units for people with SMI (predominantly schizophrenia and related psychoses) offering 24-hour intensive multidisciplinary support (30) to people who are typically admitted with treatment resistance and functional disability (31). CCU’s aim to deliver evidence-based rehabilitation interventions that improve independent functioning across a range of health domains (32). Acknowledging the recent expansion of rehabilitation aims for people with schizophrenia to include physical health interventions; understanding the differential impact of exercise types on mental health and functional outcomes in rehabilitation settings is important (33, 34).

Previous research in residential rehabilitation settings revealed feasibility and improvements in functional fitness following implementation of a supervised exercise interval circuit program comprising aerobic interval training involving both aerobic, body weight and resistance elements (24). Overall program acceptability was high, with resistance elements being a preferred component by participants of that program.

Objectives

Using an exploratory 8-week randomised, controlled trial; the primary objective in this study is to assess the feasibility and acceptability of stand-alone RT compared to control, aerobic interval training (AIT) for people with chronic psychotic disorders undergoing psychiatric rehabilitation.

Primary Objectives

To assess the feasibility and acceptability of stand-alone RT compared to control, aerobic interval training(AIT) based on recruitment, retention and participation.

Secondary Objectives

To determine if 8 weeks of stand-alone RT compared to control, (AIT) improves mental (functioning, mental health symptoms) and physical health outcomes

To investigate comparisons of psychological responses to a single session of exercise (week 3 and 8) between exercise conditions, RT and AIT

To correlate preference for exercise condition (RT or AIT) prior to randomisation with subsequent adherence.

Study Design

This study will be conducted as a single blind exploratory randomised controlled trial. Participants will be randomised to a supervised resistance training (RT) program or a supervised moderate intensity aerobic interval intervention (AIT), matched for volume and intensity, with the aim to compare RT with AIT with respect to our primary outcome (feasibility) in people with chronic psychotic disorders undergoing psychiatric rehabilitation.

**Feasibility Criteria**

Recruitment – percentage of participants who consent to the study as a proportion of CCU residents who are referred from the three sites. Recruitment into exercise trials for people with SMI can be highly variable, however > 50% (majority) will be considered feasible.

Retention – the proportion of participants consenting to the trial who complete follow up assessments at 8 weeks as a proportion of the total number allocated in each condition. Feasibility will be defined as > 65% based on previous research (49).

Participation – the number of supervised exercise sessions attended by participants in each condition (RT and AIT group) as a proportion of total available sessions (3 sessions per week for 8 weeks - 24); feasibility will be defined by attendance at greater than 60% of sessions (15 sessions).

Participants will be evaluated at baseline and end point week 8. Total duration of the study will be 8 weeks. All outcome raters will be blind to outcome allocation, however blinding of participants to their exercise condition will not be possible.

Study Population

Forty participants (50) undergoing psychiatric rehabilitation will be recruited through three Metro South Addiction and Mental Health Service Community Care Units (Logan, Coorparoo, Bayside).

Number of participants

The study will consist of a total of 50 participants.

4.2 Inclusion Criteria

Patients will be invited to participate in the study if they meet all of the following criteria:

Aged between 18-64 years (inclusive)

Fulfil the DSM 5 criteria for schizophrenia and related psychotic disorders (any psychotic spectrum disorder such as schizophrenia, schizoaffective disorder, bipolar disorder with psychotic features, major depressive disorders with psychosis) as confirmed by the treating psychiatrist of the unit.

Have a treatment plan such that they are likely to reside within the CCU for the duration of the 8-week study

Agree to participate, has capacity to consent and able to follow the study instructions and procedures.

Has been cleared to participate in an exercise program by the accredited exercise physiologist (AEP) within the service, following standard assessment of suitability and risk as per AEP exercise guidelines within the CCU’s.

Any participant deemed at risk of injury or considered medically unfit will be advised to be reviewed by their General Practitioner (G.P) and written clearance provided by the G.P before participation will be allowed in the research program (also in accordance with current policy at each CCU to allow access to the existing exercise program).

4.3 Exclusion Criteria

Potential participants will be excluded if:

They are pregnant

They have a comorbid eating disorder in which exercise is considered to be contraindicated by the treating psychiatrist of the unit (ie anorexia nervosa where BMI <18; presence of medical complications of an eating disorder; or anorexia or bulimia nervosa is comorbid with exercise addiction)

Substance abuse is considered to be interfering with rehabilitation engagement, and their ongoing suitability for rehabilitation is under review by the treating team.

Inability to follow the study instructions and procedures

4.4 Screening assessment

Researchers will work with senior nursing staff of three CCU’s for a 3-week period prior to the intervention start date to identify those residents who might meet criteria for the study. After verbal consent is provided, an assessment of inclusion/exclusion criteria will commence. Participants who meet all inclusion criteria and none of the exclusion criteria will be invited to participate in the study and the formal consent process will commence. For those who consent to participate, they will be enrolled in the study and randomized.

Prior to randomisation, all participants recruited to the study will be assessed for baseline characteristics regarding: primary and secondary mental health diagnosis using DSM V by the Consultant psychiatrist on the research team, a range of sociodemographics (age, gender, employment, education, income, ethnicity), medication use – number and type of medications and chronic health conditions. Doses of antipsychotics will be converted to chlorpromazine equivalents using standardised measures (39).

Participant Information and Informed Consent

Eligible participants (18-64 years) will be given a full explanation in lay terms, with a friend or family member present if desired, of the study aims, the discomfort, risks and benefits in taking part and a copy of the Participant Information Sheet Consent Form to review.

If the participant meets criteria to enter the study and has capacity, then informed written consent will be obtained. It will be explained to participants that they can withdraw from the study at any time without prejudice and will not affect their current care. The participant will have the opportunity to ask questions. A telephone number will be provided so that participant can call a research representative who will be able to respond to any questions they may have.

Each participant will acknowledge receipt of this information by giving written informed consent for participation in the study. A notation that written informed consent has been obtained will be made on the participant’s Case report Form (CRF). The original, completed consent forms will be retained by the Investigator and a copy will be provided by the research staff to the participants.

Study Assessments and Procedures

Trained research assistants, who are blind to group membership, will conduct all assessments.

Measures conducted *at baseline only*, prior to randomisation:

Previous exercise experiences and history, exercise preferences, barriers and enablers to exercise will be assessed using Preferences, Barriers and Motivators questionnaire (40-42). Participants will be specifically asked the type of exercise they would prefer (RT or MIIT, or no preference) prior to randomisation. Self-report exercise will be obtained via the SIMPAQ, which is a researcher-administered self-report questionnaire assessing time (average minutes and hours) spent in bed, sitting or lying down, napping during the day, walking, structured exercise, and incidental activities completed in the previous week that has recently undergone an international validation study and correlated with objective physical activity that has undergone recent validation in people with severe mental illness (43).

Participants will be asked to wear to a GENEActiv wrist-worn accelerometer, (GENEActiv, Kimbolton, UK) for 5 days at baseline to measure habitual physical activity. Accelerometers have been shown to be acceptable for people with mental illness (44) and the most preferred way for measurement of physical activity by people with SMI (45). There is scant data regarding minimum wear time in people with SMI, however prior studies in the general population indicate 3.4 days is sufficient in the general population to assess habitual physical activity (46, 47). Habitual sedentary behaviour and walking activity will be measured each day the accelerometer is worn.

The accelerometer will record how many minutes per day each participant is sedentary and engages in light, moderate and vigorous physical activities. A recording is made of each 60-second period (defined as an ‘epoch’), and this is classified as being either sedentary, light, moderate or vigorous physical activity. Cut points have been defined according to metabolic equivalents (METs) of sedentary (<1.5 METs), light (1.5–3.99 METs), moderate (4.00–6.99 METs) and vigorous (>7+ METs), based on standardised algorithms with high sensitivity and specificity with oxygen consumption (48). Habitual physical activity will be measured as time spent in physical activity (minutes per day in light, moderate and vigorous activity) and total minutes of sedentary behaviour per day.

A battery of validated clinical and physical measures will be *conducted both at baseline and endpoint, week 8*. Baseline assessments will be conducted prior to randomisation:

**Measures include:**

WHODAS 2.0 –a reliable and valid assessment instrument for health and disability in all adult populations across different cultures, which covers six domains of functioning including cognition, mobility, self -care, getting along, life activities and participation. It is a 36 item questionnaire and will be interviewer administered.

Psychiatric symptoms will be measured using the Brief Psychiatric Rating Scale (BPRS) through the use of a semi-structured interview guide and an anchored scoring version of the BPRS-A. The BPRS was developed for efficient and comprehensive assessment of change across a broad range of psychiatric symptoms, is brief, reliable and sensitive and has been used extensively across a variety of settings and patients (1, 2).

Negative symptoms will be measured by the Scale Assessment of Negative Symptoms (SANS), a reliable and valid scale, using the Interview guide for Assessment of Negative symptoms (IG-SANS) (53-55).

Motivation to exercise; The Behavioural Regulation in Exercise Questionnaire (BREQ3) is a 24-item questionnaire used to assess amotivation, and external, introjected, identified, integrated and intrinsic behavioural regulations for exercise (four questionnaire items for each behavioural regulation subscale). Scoring is on a 5-point Likert scale ranging from 0 ('Not true for me') to 4 ('Very true to me'). BREQ-3 has high test-retest reliability (ρ>0.7) and has been shown to be moderately predictive of exercise participation (56).

Physical capacity will be measured using three tests; the six-minute walk test (6MWT), which is a submaximal test to assess functional capacity that is commonly used for adults with mental illness (3). The 6MWT involves the participant walking as far as possible back and forth along a flat 20 m surface in six minutes; the tester provided standardised encouragement every minute consistent with guidelines (4).

The 30-second sit-to-stand-to-sit test will be used to assess lower body strength, for which participants were asked to stand up from a seated position and sit back down as many times as possible without using their arms in 30 seconds. The 30 second test has no ceiling and hence can be used in participants with a wide variety of ability levels. This test has been shown to correlate well with a 1-RM (one repetition maximum) leg-press test in community dwelling older adults (r>0.7), and hence suitable for our untrained participants, and has good test-retest reliability (59).

Grip Strength will be measured using a dynamometer. Maximal isometric handgrip is considered suitable for a variety of clinical settings and provides a simple and valid proxy of upper body muscular strength (19, 60).

Strength will be measured using the 1 minute AMRAP push up test – in which participants are asked to perform as many push ups as they can in one minute. At baseline, participants may choose to perform push ups from the knees, as a full body push up, or against a wall incline, depending on baseline conditioning. The push up test will be repeated in week 8 as the same format as was selected at baseline, ie if they select full body push ups at baseline, this will be repeated at week 8 (5).

Physical health measures will include; blood pressure will be taken manually with a sphygmomanometer in a seated position after 5 min of rest. An appropriate cuff size will be selected for each participant based on arm circumference. Height will be measured to the nearest 0.1 cm using a stadiometer, and weight will be measured to the nearest 0.01 kg using electronic scales (Charder MS 6111). Waist circumference will be measured at the mid-point between the iliac crest and lower ribs to the nearest 0.1 cm using a tape measure that provides 100g of tension. Body Mass Index (BMI) will be calculated (weight/health2).

Subjective Experience of Exercise Scale (SEES) (61) in week 3 (once competence has developed) and week 8 (at the end of the intervention). The SEES consists of 12 items that measure global psychological responses to the stimulus properties of exercise with items being determined to sit on three domains – psychological wellbeing, psychological distress and fatigue. The three subscales have been determined to have good internal consistency (62).

Strength – the 12RM will be measured pre and post the intervention in large muscle groups – squat and chest press, in the RT group only (due to the complexity of the testing).

Following *completion of the trial, acceptability* will be assessed:

Participants will be asked to complete a short self-report questionnaire regarding their exercise experiences at the end of the 8-week intervention. Responses will be reported on a 5 point Likert scale, with answers from Strongly disagree, disagree, neutral, agree and strongly agree.

For those willing to participate, acceptability will also be assessed via qualitative interviews. Participants from the intervention condition (Resistance Training, RT) will be invited to engage in an individual 20-minute semi-structured interview with a researcher not involved in the delivery of the intervention. The interview will cover topics including likes, dislikes, effectiveness and recommendations for future iterations. For those participants who consent Interviews will be audio-recorded on a dictaphone and uploaded to a password protected Queensland health computer. They will be de-identified and sent to a professional transcription company to be transcribed into written content, coded as interviewer and interviewee. Data will be analysed using thematic analysis to determine key themes identified by participants (6).

**Table 1:**

| **Visit** | 0  Screening  Phase | 1  Baseline | 2 | 3 | 4 | 5 | 6 | 7 | 8 | 9 |
| --- | --- | --- | --- | --- | --- | --- | --- | --- | --- | --- |
| **WEEK** |  | 0 | 1 | 2 | 3 | 4 | 5 | 6 | 7 | 8 |
| **SCREENING AND CONSENT** |  |  |  |  |  |  |  |  |  |  |
| Informed consent | x |  |  |  |  |  |  |  |  |  |
| Ongoing capacity | x |  |  |  |  |  |  |  |  |  |
| Inclusion / exclusion criteria | x |  |  |  |  |  |  |  |  |  |
| Baseline demographics | x |  |  |  |  |  |  |  |  |  |
| **SAFETY** |  |  |  |  |  |  |  |  |  |  |
| Adverse events (before/after each session by the AEP) |  |  | x | x | x | x | x | x | x | x |
| **EFFICACY** |  |  |  |  |  |  |  |  |  |  |
| Accelerometry (worn for 5 days prior to randomisation) |  | x |  |  |  |  |  |  |  |  |
| SIMPAQ |  | x |  |  |  |  |  |  |  |  |
| Preferences, Barriers, Motivators Questionnaire |  | x |  |  |  |  |  |  |  |  |
| WHO DASS 2.0 |  | x |  |  |  |  |  |  |  | x |
| BPRS |  | x |  |  |  |  |  |  |  | x |
| SANS |  | x |  |  |  |  |  |  |  | x |
| BREQ 3 |  | x |  |  |  |  |  |  |  | x |
| Physical Capacity (6MWT, 30 sec sit-to-stand, grip strength, push up test) |  | x |  |  |  |  |  |  |  | x |
| Physical Health measures (BP, height, weight, waist circumference) |  | x |  |  |  |  |  |  |  | x |
| 12RM strength, chest press and squat(RT only) |  | x |  |  |  |  |  |  |  | x |
| SEES |  |  |  |  | x |  |  |  |  | x |
| **INTERVENTION SESSIONS** |  |  |  |  |  |  |  |  |  |  |
| RT and AIT (x3 sessions per week) |  |  | x | x | x | x | x | x | x | x |
| Health Coaching Sessions offered |  |  | x |  | x |  | x |  | x |  |
| **ACCEPTABILITY** |  |  |  |  |  |  |  |  |  |  |
| Acceptability questionnaire |  |  |  |  |  |  |  |  |  | x |
|  |  |  |  |  |  |  |  |  |  |  |
| Individual interview (RT condition) - optional |  |  |  |  |  |  |  |  |  | x |

**Schedule of Visits and Assessments**

Study Restrictions

For the period of this study, participants will be asked not to engage in exercise outside of the exercise type they have been randomised to, outside of supervised study sessions.

**Safety Assessments**

All patients recruited in this study will be active cases at Metro South Addiction and Mental Health Service Community Care Units. The study team will liaise with clinical staff to ensure that there are no unforeseen contraindications to the participant being involved.

Both resistance training and aerobic interval training are considered safe and feasible for people with severe mental illness, and all sessions will be fully supervised by an AEP (7, 8).

Adverse Events

Data on adverse events in both conditions will be reported, including any new injuries, exacerbations of pre-existing conditions, participant withdrawal and reasons and adverse events judged to be as a direct result of the intervention will be specifically recorded in the case report form. We will examine adverse events at before and after each training session. All adverse events reported between consent and final visit will be recorded in the case report form (CRF). The investigator or AEP will ask the Participant non-leading questions in an effort to detect adverse events e.g. “How have you been over since the last session”. We expect that adverse events will occur during the study related to fluctuations in the underlying mental disorder and medications and adjustment to increased physical activity.

Investigational Exercise(s)

Description of Investigational Exercise(s)

The study will be conducted onsite at each CCU, within the shared courtyard, using pre-existing gym facilities and equipment maintained by each CCU. Both resistance training and AIT will be conducted in accordance with recommendations from the Sports Science Checklist (Supplementary material 1), in order to improve both the quality of reporting, and of methodological aspects of training and fitness assessments.

Both conditions will offer engagement in a total volume of moderate intensity exercise that will be greater than 90 min per week, which has been shown to be associated with improvements in mental health outcomes (13). Both exercise conditions (RT and control) will be aimed at moderate intensity exercise that will be balanced for total number of sessions and volume. The total volume of activity in RT was estimated to match what would have been attained if assigned to the AIT and in line with previous research protocols comparing aerobic with RT interventions in this population.

**7.1.1 Resistance training Intervention (RT)**

The World Health Organisation and Australian Physical Activity Recommendations suggest resistance training to occur on 2 or more days per week, involving main muscle groups (9, 10).

The intervention will involve resistance training three days per week, using a variety of resistance training equipment such as hand weights and resistance bands appropriate for small clinical gym settings to enable replication of the study without substantial investment in specialised equipment or gym spaces.

There will be a 10 minute stretching warm up and 5 minute cool down. The resistance training intervention will take approximately 30 minutes duration, with total duration 45 minutes. The active exercise volume will be progressed to 40 minutes by week 4 of the intervention, with a total duration of exercise being 50-55 minutes.

Resistance training will be progressive and tailored to the individual participant’s current ability/ exercise capacity and exercise history via individually prescribed programs, taking into account participants with schizophrenia who may be deconditioned and may have little experience with resistance training. At all times training will be tailored to their experience level by experienced AEP’s trained exercise prescription. An initial assessment of muscle strength will be assessed using the 12 repetition maximum test (12-RM) for relevant large muscle groups to guide exercise prescription, which is an appropriate proxy measure to estimate the 1-RM (1 Repetition Maximal test) in poorly conditioned individuals, using an established conversion formula; Brzycki 1RM Formula, 1RM = Weight x (36 / (37 – Repetitions),(11).

The aim will be to increase muscle strength progressively over the 8 weeks. Training will be appropriate for beginners and be aimed at moderate intensity (40-70%) of estimated maximum strength, (1-RM). Week 1 will start with 40% of 1-RM, and progressed with 5% increments each week as per capacity of the individual, with 3 sets of 8-10 repetitions, of approximate 40 seconds duration per set, and a 90 seconds duration between sets.

Six exercises across main muscle groups used in activities of daily living will be prescribed per resistance training session. As strength increases, sessions will be progressed using heavier weights, however each participant will be monitored for their response to training, and progression individualised. A typical session will include 6 exercises that target main muscle groups such as lower legs, back, arms/shoulders, and core, using a variety of standard resistance training equipment, for example; hand weights, kettle bells, resistance bands and a chest press bar and floor mat. Exercises will include Romanian and sumo squats, forward lunges, bicep curls, tricep extensions, calf raises, reverse fly, shoulder press and crunches. All exercises are appropriate for resistance training in untrained participants and will be supervised by the AEP employed at each site. The content and weights of each session will be recorded using standard protocol.

**7.1.2 Aerobic Interval Training (AIT)**

Participants randomised to the aerobic interval intervention will engage in a 10 minute warm up and 5 minute cool down and a 30 minute aerobic interval training intervention in a circuit style, with total duration of exercise 45 minutes. The active exercise volume will be progressed to 40 minutes by week 4 of the intervention, with a total duration of exercise being 50-55 minutes.

Equipment used for the circuit will include a stationary bike, a treadmill, boxing pads and gloves, step and mat. The circuit stations will vary each week to reduce boredom. Stations are designed such that they could be modified (regressed/progressed) to suit individual participant capacity and preference.

Participants will engage in a circuit style training supervised by the AEP at each site, three times per week. The aerobic intervention will begin slowly, aimed at beginners, but adapted for experience level and baseline fitness of participants.

Intensity of the aerobic intervention will be monitored using the Borg Category Ratio 10 scale of Perceived Exertion scale (RPE) (24), a 10 point scale to assess subjective perception of effort that has been calibrated against exercise intensity and will guide intensity of exercise prescription. Initial prescription will be aimed at an intensity achievable for participants with low initial CRF but progressed to reach moderate intensity by week 3 (12). The AEP will aim for the participant to maintain an RPE at 2–3 for the first 2 weeks, then increased to a minimum RPE of 4/10 by week 3 and further increased by 5% each week as per individual participant's capacity. Following warm-up, participants will rotate through interval stations, beginning with four minutes of exercise interspersed with three minutes of active recovery (reduced intensity interval RPE 1-2) until a total of 30 minutes of MIIT is completed. Participants will progress to five minute active intervals as the participants' functional capacity increases.

**7.1.3 Fidelity**

A one day training program for AEP’s at each CCU will be conducted in order to train them in the specific delivery of the intervention such that participant programs will be standardised for each condition and conducted in accordance with the protocol. Programs will be developed for individuals by each AEP at the CCU and reviewed by the AEP from the research team each week to ensure sessions from each condition are conducted within the scope of the intervention. This will include; terms of number of repetitions, progression, and any necessary adaptation for pre-existing musculoskeletal conditions if present.

**7.1.4 Strategies to enhance adherence**

Participants from each condition will be offered a 20 minute fortnightly health coaching session utilising evidence based motivational strategies such as self- monitoring, goal setting, developing an understanding the value of physical activity and building self-efficacy for exercise, that is usual practice for accredited exercise physiologists. Motivational strategies will be based on Self Efficacy Theory which has been utilised in previous exercise interventions for people with SMI (13). Motivational strategies are recommended to increase adherence to exercise interventions (8). Participants will be encouraged to play music of their choice whilst exercising to enhance pleasure, or to choose to watch youtube videos on the CCU laptop. Participants will also be able to choose whether they wish to engage in sessions individually or in small groups, and to choose preferred exercise equipment or type of resistance activities within an accepted range of available exercises to ensure personal preference and autonomy is maximised, despite randomisation to condition.

**7.1.5 Randomisation Procedure and blinding**

Participants will be randomised once written consent has been obtained and the baseline assessments have been completed.

Participants will be assigned a unique code that will be sent to an independent biostatistician for randomisation using a computer -generated sequence of numbers. Randomisation to either RT or MIIT condition, will occur in a 1:1 ratio, using block randomisation.

Once randomisation has occurred, participants will be notified of their group allocation by an independent staff member to ensure the research team remains blinded to allocation.

There will be no opportunity to change conditions once randomised, however if participants wish they may withdraw from the study and continue to exercise using the equipment and AEP at each CCU independently from the study. If withdrawal occurs, reasons for doing so will be recorded in the CRF. Following randomisation, participants will be asked whether they are happy with the condition they have been randomised to, and this will be recorded in the CRF.

Adverse Events (AE) and Serious Adverse Events (SAE)

The investigator is responsible for the detection and documentation of events meeting the criteria and definition of an adverse event (AE) or a serious adverse event (SAE) as provided in this protocol. During the study, when there is a safety evaluation, the investigator or site staff will be responsible for detecting AEs and SAEs, as detailed in this section of the protocol

Definition of an Adverse Event (AE)

An AE can therefore be any unfavourable and unintended sign (including an abnormal laboratory finding), symptom, or disease (new or exacerbated) temporally associated with the use of a medicinal product, whether or not considered related to the medicinal product. For marketed medicinal products, this also includes failure to produce benefits (i.e. lack of efficacy), abuse or misuse.

Examples of an AE **include**:

Acute episode of psychosis

Exercise induced injury or complaint

Examples of an AE **do** **not include** a/an:

Medical or surgical procedure (e.g. endoscopy, appendectomy); the condition that leads to the procedure is an AE.

Situations where an untoward medical occurrence did not occur (social and/or convenience admission to hospital).

In this study, AEs may include pre- or post-intervention events that occur as a result of protocol-mandated procedures (i.e. invasive procedures, modification of participants’ previous therapeutic regimen).

Definition of a Serious Adverse Event (SAE)

A serious adverse event is any untoward medical occurrence that, at any dose:

a) results in death

b) is life threatening

c) requires hospitalisation or prolongation of an existing hospitalisation.

d) results in disability/incapacity, or

f) Any event deemed by the investigator as being a significant medical event.

Time Period, Frequency, and Method of Detecting AEs and SAEs

All adverse events will be recorded between the time of consent and the follow-up visits. Each Participant will be monitored regularly by the investigator and study personnel for adverse events occurring throughout the study. The research team will enquire about AEs by asking the following non-leading questions:

At the beginning and post intervention participants will be asked:

*“How are you feeling?” Does your current intervention cause you regular side effects? Do you have any general health conditions that cause you problems on a regular basis (e.g. that we might expect to occur over the duration of this study?"*

At subsequent scheduled visits, participants will be asked:

*“Since your last visit, have you had any health problems?”*

Recording of AEs and SAEs

If an AE/SAE occurs during the course of the intervention the Investigator/delegated research staff will report accordingly with immediate medical attention sought as per usual CCU guidelines for the safe prescription of exercise within the facilities and record all relevant information regarding an AE/SAE in to the CRF. Exercise may be able to be modified to allow continued participation in the program for any minor injuries or exacerbations of pre-existing conditions, following review by a General Practitioner and with the consent and agreement of the participant and research team (and as per usual exercise injury management policy of the CCU).

Evaluating AEs and SAEs

Assessment of Intensity

The investigator will make an assessment of intensity for each AE and SAE reported during the study. The assessment will be based on the investigator’s clinical judgement. The intensity of each AE and SAE recorded in the CRF should be assigned to one of the following categories:

**Mild:** An event that is easily tolerated by the Participant, causing minimal discomfort and not interfering with everyday activities.

**Moderate:** An event that is sufficiently discomforting to interfere with normal everyday activities.

**Severe:** An event which is incapacitating and prevents normal everyday activities.

An AE that is assessed as severe should not be confused with an SAE. Severity is a category utilised for rating the intensity of an event; and both AEs and SAEs can be assessed as severe. An event is defined as “serious” when it meets one of the pre-defined outcomes as described in Section 8.2 “Definition of an SAE”.

Assessment of Causality

The investigator is obligated to assess the relationship between the intervention and the occurrence of each AE/SAE. The investigator will use clinical judgment to determine the relationship. Alternative causes, such as natural history of the underlying diseases, concomitant therapy, other risk factors, and the temporal relationship of the event to the intervention will be considered and investigated.

The causal relationship to the study product assessed by the Investigator (or medically qualified delegate) should be assessed using the following classifications:

**Not Related** In the Investigator’s opinion, there is not a causal relationship between the study intervention and the adverse event.

**Unlikely** The temporal association between the adverse event and study intervention is such that the study intervention is not likely to have any reasonable association with the adverse event.

**Possible** The adverse event could have been caused by the study Participant’s clinical state or the study intervention.

**Probable** The adverse event follows a reasonable temporal sequence from the time of study intervention, abates upon discontinuation of the study intervention and cannot be reasonably explained by the known characteristics of the study Participant’s clinical state.

**Definitely** The adverse event follows a reasonable temporal sequence from the time of study intervention or reappears when study intervention is reintroduced.

Follow-up of AEs and SAEs

All AEs and SAEs documented at a previous visit/contact and are designated as ongoing, will be reviewed at subsequent visits/contacts.

All AEs and SAEs will be followed until resolution, until the condition stabilises, until the event is otherwise explained, or until the Participant is lost to follow-up. Once resolved, the appropriate AE/SAE CRF page(s) will be updated.

8.7 Risk Management Process

Table 2 below details the Risk Identification, Evaluation and Management plan for this study.

It will ensure that risk and uncertainly are appropriately managed for the duration of the study. The risk management process is in accordance with the NHMRC National Statement on Ethical Conduct in Research Involving Humans (2007).

**Table 2: Risk Analysis Matrix**

| **Consequence** | **Response To Risk** |
| --- | --- |
| \| **Likelihood** \| **Negligible** \| **Minor** \| **Moderate** \| **Major** \| **Extreme** \| \| --- \| --- \| --- \| --- \| --- \| --- \| \| Almost Certain \|  \|  \|  \|  \|  \| \| Likely \|  \|  \|  \|  \|  \| \| Possible \|  \|  \|  \|  \|  \| \| Unlikely \|  \|  \|  \|  \|  \| \| Rare \|  \|  \|  \|  \|  \| | \|  \| Very High \| Immediate action required \| \| --- \| --- \| --- \| \|  \| High \| Urgent attention or investigation required \| \|  \| Medium \| Require specific attention \| \|  \| Low \| Manage through routine procedures \| |

**Risk Identification, Evaluation and Management Plan**

|  | Risk | Description | Possible Effects | | | Risk Management strategies |
| --- | --- | --- | --- | --- | --- | --- |
|  |  |  | Likelihood | Consequence | Rating |  |
| 1. | Psychological discomfort during assessments | Participants may experience psychological discomfort when answering questions in the clinical interview and cognitive assessments. | Possible | Minor-moderate | Medium | The PICF clearly states the potential risk of discomfort.  Recruitment of experienced mental health clinicians who will be able to minimise and manage discomfort.  Participants will be clinically assessed at baseline, 3 weeks and at 8 weeks. Participants are given the opportunity to discuss any concerns/discomforts re previous appointment.  Clinicians will direct and assist participants to gain support if required. |
| 2. | Inconvenience of participating in the trial | Participants may be inconvenienced by time taken to participate in the trial. | Possible | Negligible | Low | The PICF clearly states the battery of clinical assessments and physical interventions to be completed and the approximate time and frequency for clinical assessment and physical intervention visits.  Participants will be given as many breaks as necessary throughout the clinical assessment visit.  Participants will be provided with either baseline and post intervention gift cards, or Garmin Vivofit wearable device for their time involved in the trial.  Participants will be reminded that the trial is voluntary and they can withdraw at any time. |
| 3. | History of self-harm/suicidal ideation | Participant expresses suicidal ideation. | Possible | Moderate-severe | High | Recruitment of experienced mental health clinicians who are trained in conducting risk assessment and managing high risk situations.  Research staff will have access to a clinically trained senior staff and Chief Investigator who will assist research staff to conduct risk assessment and implement risk management plan if required i.e. notifying treating team and assisting in the participant accessing appropriate support (e.g. emergency services)  Research staff will be given support and feedback on risk assessments and their management to improve skills throughout the project.  Residents of CCU’s must be low imminent risk of suicide and aggression to be able to reside in residential facilities and are screened for risk by the admitting consultant psychiatrist before admission. |
| 4. | Physical discomfort | Participant experiences an injury during the intervention | Possible | Minor – moderate | Medium | AEP will be conducting the intervention and will monitor participants for any type of injury or physical discomfort and treat and report according to the facilities policy and procedures.  The interventions will be tailored to take into consideration the participant’s level of fitness. |

Participant Completion and Discontinuation

Participant Completion

Participants are considered to have completed the study if they complete 8 weeks of intervention. The number of supervised exercise sessions attended by participants in each condition (RT and MIIT group) as a proportion of total available sessions (3 sessions per week for 8 weeks - 24); feasibility will be defined by attendance at greater than 60% of sessions (15 sessions).

Participants will be asked to complete post measures even if they are withdrawn from the study intervention, where possible.

Participant Withdrawal by the Investigator

Worsening of mental state such that the patient is admitted to hospital or their ability to provide ongoing informed consent is compromised or physical injury preventing the participant from continuing in the clinical trial.

Case Report Form (CRF)

A Case Report Form (CRF) will be completed for each study participant summarising all clinical screening and study data that will be used by the research team for analysis. In the CRF, participants will only be identified by their participant number in order to retain participant confidentiality.

The completed Case report Forms (CRF’s) will be retained by the Investigators for a period of at least 15 years or the maximum time frame as determined by local regulations, whichever is the longest.

Data Analysis and Statistical Considerations

Hypotheses

We hypothesise that stand alone RT will be feasible and acceptable to people with psychotic disorders accessing residential rehabilitation.

Endpoints

Primary

Stand-alone RT will be feasible and acceptable compared to control, aerobic interval training (AIT) based on recruitment, retention and participation.

Secondary

Comparison of stand-alone RT with control, aerobic interval training(AIT) with respect to mental (functioning, mental health symptoms) and physical health outcomes

Allocation to exercise condition the participant expresses preference for prior to randomisation will result in increased adherence.

Comparison of psychological responses to a single session of exercise (week 3 and 8) between exercise conditions, RT and MIIT will be identified

Sample Size and Power

This study will be conducted as an exploratory RCT with feasibility as the primary outcome. Based on recruiting an adequate sample to assess the feasibility of the study with the resources available (63) we aim to recruit 50 participants assuming approximately 25 participants will be assigned to each intervention arm, based on previous exercise research within this setting – we anticipate 20% drop outs per exercise condition (49).

Statistical Analysis

Data analyses will be conducted using SPSS Statistics 24, (SPSS Inc, Chicago, Illinois) for the primary outcomes. Continuous variables were summarized using means and standard deviations (SD) (or median and interquartile range for non-parametric data). All variables will be tested for normality using the Shapiro-wilks test, and visual inspection of Q-Q plots. Baseline demographic and clinical variables, participation and AE rates and mean aggregate acceptability scores will be compared between the two exercise groups using independent t-tests or Mann-Whitney U for non-parametric data. Chi square analysis for categorical data will be used to detect any baseline differences between the two exercise groups and for comparison of acceptability categories (strongly agree/agree versus other) per questionnaire item between the groups.

Baseline demographic and clinical variables will be compared between the 2 exercise groups using independent t tests (or Mann Whitney U tests if data non parametric) for continuous data and Chi2 square analysis for categorical data to detect any baseline differences between the 2 exercise groups. Baseline characteristics of those who complete >50% of the program will be compared to those who withdraw to identify characteristics of those who drop out.

To analyse the impact of the proposed exercise intervention on secondary outcomes, analyses will be performed on an intention-to-treat basis. Change from baseline in secondary outcomes at week 8 will be calculated as least squares (LS) means using a Linear Mixed Effects Model (LMM). Treatment group (RT or AIT) and time will be used as factors, with covariates established in previous similar research (age, gender, participation, and antipsychotic medication use),(García-Garcés et al., 2021b). Where group/time differences were detected, posthoc (Bonferroni) analyses will be carried out separately for the intervention and control group, with Cohen’s d estimate of effect sizes provided. LMM was performed using R (software version 4.04, 2024). All the statistical tests were two-tailed with the critical P value for significance set at?<?0.05.

For the RT group only, 12 RM strength will be assessed for normality and compared pre and post the intervention using paired samples t test.

To examine the association between exercise preference prior to randomisation with participation, a one-way ANOVA compared exercise participation rates between three groups; (i) those who randomly received their exercise preference, (ii) those who did not, (iii) those who expressed no exercise intervention preference.

The three feeling state domains from the SEES would be assessed using a group (RT, MIIT), time (pre/post) ANOVA with repeated measures. The effect size of the differences between values will be interpreted using Cohen’s *d* statistic ([Cohen, 1988](https://journals-humankinetics-com.ezproxy.library.uq.edu.au/view/journals/japa/27/3/article-p325.xml#r8)). An effect size of <0.2 reflects a negligible difference; ≥0.2 to ≤0.5, a small difference; ≥0.5 to ≤0.8, a moderate difference; and ≥0.8, a large difference.

Data Management

A screening log will be utilized to track potential participants and also record the counts of individuals approached, consented, meeting inclusion/exclusion criteria, withdrawals, and completion (in keeping with standard CONSORT diagram requirements).

The Case Report Form (CRF) will comprise of the hard copy questionnaires, clinical assessments and measures. These de-identified data will be retained in a secure room, in a locked filing cabinet at Queensland Centre for Mental Health Research.

De-identified data from the CRFs will be entered into a spreadsheet that is stored on a password protected Queensland Health computer, which only members of the research team will have access to. Delegated research assistant will be trained in, and responsible for, entering data into the database. A copy of the PICF will be stored in a secure room in a locked filing cabinet separate from the CRFs at Queensland Centre for Mental Health Research.

All data will be exported into SPSS software to enable statistical analysis.

GENEActiv accelerometer data will be downloaded at completion of baseline and analysed in Matlab 2016a (The MathWorks, Inc., Natick, Massachusetts, United States) on a password protected Queensland Health computer.

Monitoring and Quality Assurance

The investigator will submit to the Reviewing HREC, annual (or more frequent if requested) reports of the study.

The investigator will monitor data entered and be responsible for resolving data entry errors and discrepancies.

Data quality will be ensured by performing data entry checks for consistency between the CRF and the data entry into the password protected spreadsheet. These checks will be performed during data entry so that discrepancies can be resolved immediately.

Investigator Responsibility

The Coordinating Principal Investigator will be responsible for the conduct of all aspects of the study. The study and its associated documents will be reviewed and approved by the appointed certified HREC and Research Governance before study start.

Prior to submission to appointed HREC and Research Governance, the investigator will sign the protocol signature page confirming her agreement to conduct the study in accordance with the protocol, GCP and other regulatory requirements locally applicable. All relevant data and records will be provided to the HREC as required.

Each Investigator will comply with the local regulations regarding clinical trials and the Investigator responsibilities outlined in the ICH GCP guidelines.

Study Report

The Investigator will submit at least annual study reports to the reviewing HREC, or more frequent if required.

Administrative Procedures

Ethical Considerations

All documentation pertaining to the study must be prepared in accordance with the requirements outlined by the relevant ethics committee. All documentation must then be approved or given a favourable opinion in writing by an HREC as appropriate.

The Coordinating Principal Investigator is responsible for informing the HREC of any amendment to the protocol in accordance with local requirements. In addition, the HREC must approve all advertising used to recruit participants for the study.

The study will be performed in accordance with ethical principles that have their origin in the Declaration of Helsinki and are consistent with ICH/Good Clinical Practice, applicable regulatory requirements. The NHMRC National Statement on Human Research will also be utilised.

Ethical Review Committee

The HREA and associated documents will be submitted for approval to the appointed HREC and written approval obtained from both the appointed HREC and Governance Office, before volunteers are recruited and participants are enrolled. The Coordinating Principal Investigator will submit the HREA and associated documents including Site Specific Applications to the appointed HREC and Research Governance. The Coordinating Principal Investigator has overall responsibility to ensure all reports are submitted in line with the appointed HREC reporting requirements.

Informed Consent

Our criteria will ensure that recruited participants will be sufficiently competent to consent and participate in the study or to refuse consent. Current research provides evidence that while psychotic symptoms may be present, these do not robustly predict an individual’s functionality in daily life and capacity to make decisions, and whilst strongly correlated with cognitive impairment, do not reflect an enduring inability to understand information related to research participation.

Participant Reimbursement

Participants will be reimbursed for out of pocket expenses, inconvenience and time involved by the provision of either prepaid gift cards (e.g. Coles-Myer, K-Mart etc) or a Garmin Vivofit wearable device (value ~ $80) at the beginning of the intervention, the choice will be theirs. We will provide a $20 gift card to those who verbally assent to complete the SEES questionnaire before and after a single session of exercise in week 3 of the intervention. We will provide a $40 gift card on completion of the baseline assessment and at the end of study assessment (total reimbursement $100). If the study is terminated by the Investigator prior to completion, or a participant withdraws or is withdrawn from the study before completion, a pro-rata payment will be made at the discretion of the Investigator.

Participants will be given an opportunity to participate in a small group interview at the end of the intervention and will be reimbursed a $20 gift card for inconvenience and time involved.

Investigator Indemnification

The clinical trial insurance will reimburse participants for costs of medical care that occur as a result of complications directly related to participation in this study. The Investigator and insurance company will be notified as soon as possible if this occurs or where a causal relationship cannot be excluded. All SAE’s will be reported to the nominated Hospital and Health Service insurance company.

Publication Policy

Results will be disseminated in peer reviewed publications and published in international journals. Only group data will be reported.

Protocol Amendments

Any amendments to the protocol will be submitted to the appointed HREC by the Coordinating Principal Investigator for approval. Any approved amendments by the appointed HREC will be forwarded by the Coordinating Principal Investigator for submission to each Research Governance Office.

No changes (amendments) to the Protocol will be implemented without prior approval from the Reviewing Ethics Committee. If a Protocol amendment requires changes to the Informed Consent Form, the revised Informed Consent Form, prepared by the Coordinating Principal Investigator, will be approved by the Reviewing Ethics Committee and site governance officers.

Once the final Protocol has been issued and signed by the Coordinating Principal Investigator and the authorised signatories, it will not be informally altered. All protocol amendments will pass through appropriate approval steps before being implemented. Any change to the protocol constitutes an amendment.

Where the amendment affects the ongoing suitability of the study at a participating site, Research Governance approval will also be sought. The Research Governance Office will determine the ongoing suitability based on the amendment submitted.

The Coordinating Principal Investigator will submit the amendment to the appointed HREC for their approval; written approval will be obtained. Completed and signed Protocol amendments will be circulated to all appointed site Investigators.

Version Control

Version control ensures that amendments to documents are tracked and verifiable and that the correct version of a document is in use according to the relevant ethical, regulatory or local approval.

All documents will be given a version number and date e.g. Version 1.0 15-Feb-15

Each amendment to a document will require a version number and date to be updated.

If this is a **significant change** e.g. change in the content of the document, then the version number will be increased by 1.0.

If it is a **minor change** e.g. contact details, then the number after the decimal point will be increased by 0.1.

Protocol Compliance

Should there be questions or consideration of deviation from the Protocol, clarification will be sought from the Coordinating Principal Investigator. Any participant treated in a manner that deviates from the Protocol, or who is admitted into the study but is not qualified according to the Protocol, will be ineligible for analysis.

If an emergency occurs that requires a departure from the Protocol, the nature and reasons for the Protocol violation/deviation will be recorded in the CRF and the Coordinating Principal Investigator will notify the Reviewing HREC and /or Governance Office as soon as possible.

Whilst the Coordinating Principal Investigator has overall responsibility for the conduct of the study, the appointed site Investigators will have the responsibility to ensure all study personnel at their sites comply with GCP, National Statement on Ethical Conduct (2007), Australian Code for the Responsible Conduct of Research and local policies and procedures.

Archives: Retention of Study Records

All Case report Forms (CRF’s) and study documentation will be kept by the Investigators for at least 15 years or the maximum time frame as determined by local regulations, whichever is the longest.

References

1. Saha S, Chant D, Welham J, McGrath J. A Systematic Review of the Prevalence of Schizophrenia (The Prevalence of Schizophrenia). PLoS Medicine. 2005;2(5):e141.

2. Charlson FJ, Ferrari AJ, Santomauro DF, Diminic S, Stockings E, Scott JG, et al. Global Epidemiology and Burden of Schizophrenia: Findings From the Global Burden of Disease Study 2016. Schizophrenia bulletin. 2018;44(6):1195-203.

3. Jääskeläinen E, Juola P, Hirvonen N, McGrath JJ, Saha S, Isohanni M, et al. A Systematic Review and Meta-Analysis of Recovery in Schizophrenia. Schizophrenia Bulletin. 2013;39(6):1296-306.

4. Rummel - Kluge C. Negative symptoms are prevalent in antipsychotic-treated adult outpatients with schizophrenia spectrum disorders. Evidence - Based Mental Health. 2010;13(4):106.

5. Nielsen RE, Levander S, Kjaersdam Telléus G, Jensen SOW, Østergaard Christensen T, Leucht S. Second‐generation antipsychotic effect on cognition in patients with schizophrenia—a meta‐analysis of randomized clinical trials. Acta Psychiatrica Scandinavica. 2015;131(3):185-96.

6. Arango C, Garibaldi G, Marder SR. Pharmacological approaches to treating negative symptoms: A review of clinical trials. Schizophrenia Research. 2013;150(2-3):346-52.

7. Vancampfort D, Rosenbaum S, Schuch F, Ward P, Richards J, Mugisha J, et al. Cardiorespiratory Fitness in Severe Mental Illness: A Systematic Review and Meta-analysis. Sports Medicine. 2017;47(2):343-52.

8. Stubbs B, Williams J, Gaughran F, Craig T. How sedentary are people with psychosis? A systematic review and meta-analysis. Schizophrenia Research. 2016;171(1-3):103-9.

9. Vancampfort D, Probst M, De Herdt A, Corredeira RMN, Carraro A, De Wachter D, et al. An impaired health related muscular fitness contributes to a reduced walking capacity in patients with schizophrenia: a cross-sectional study.(Research article)(Report). BMC Psychiatry. 2013;13:5.

10. Vancampfort D, Probst M, Scheewe T, Knapen J, De Herdt A, De Hert M. The functional exercise capacity is correlated with global functioning in patients with schizophrenia. Acta Psychiatrica Scandinavica. 2012;125(5):382-7.

11. Stubbs B, Vancampfort D, Hallgren M, Firth J, Veronese N, Solmi M, et al. EPA guidance on physical activity as a treatment for severe mental illness: a meta-review of the evidence and Position Statement from the European Psychiatric Association (EPA), supported by the International Organization of Physical Therapists in Mental Health (IOPTMH). European Psychiatry. 2018;54:124-44.

12. Dauwan M, Begemann MJH, Heringa SM, Sommer IE. Exercise Improves Clinical Symptoms, Quality of Life, Global Functioning, and Depression in Schizophrenia : A Systematic Review and Meta-analysis. 2016.

13. Firth J, Cotter J, Elliott R, French P, Yung AR. A systematic review and meta-analysis of exercise interventions in schizophrenia patients. 2015;45(7):1343-61.

14. Martin H, Beard S, Clissold N, Andraos K, Currey L. Combined aerobic and resistance exercise interventions for individuals with schizophrenia: A systematic review. Mental Health and Physical Activity. 2017;12:147-55.

15. Caspersen CJ, Powell KE, Christenson GM. Physical activity, exercise, and physical fitness: definitions and distinctions for health-related research. Public health reports (Washington, DC : 1974). 1985;100(2):126-31.

16. Ashdown-Franks G, Firth J, Carney R, Carvalho AF, Hallgren M, Koyanagi A, et al. Exercise as Medicine for Mental and Substance Use Disorders: A Meta-review of the Benefits for Neuropsychiatric and Cognitive Outcomes. Sports Medicine (Auckland, NZ). 2019;50(1).

17. Keller-Varady K, Varady PA, Röh A, Schmitt A, Falkai P, Hasan A, et al. A systematic review of trials investigating strength training in schizophrenia spectrum disorders. Schizophrenia Research. 2018;192:64-8.

18. Leong DP, Teo KK, Rangarajan S, Lopez-Jaramillo P, Avezum A, Orlandini A, et al. Prognostic value of grip strength: findings from the Prospective Urban Rural Epidemiology (PURE) study. The Lancet. 2015;386(9990):266-73.

19. Firth J, Stubbs B, Vancampfort D, Firth JA, Large M, Rosenbaum S, et al. Grip Strength Is Associated With Cognitive Performance in Schizophrenia and the General Population: A UK Biobank Study of 476559 Participants. Schizophrenia Bulletin. 2018;44(4):728-36.

20. Kelley AG, Kelley SK. Progressive Resistance Exercise and Resting Blood Pressure: A Meta-Analysis of Randomized Controlled Trials. Hypertension: Journal of the American Heart Association. 2000;35(3):838-43.

21. Stanton R, Reaburn P, Happell B. Is cardiovascular or resistance exercise better to treat patients with depression? A narrative review. Issues in mental health nursing. 2013;34(7):531.

22. Andrade E Silva B, Cassilhas RC, Attux C, Cordeiro Q, Gadelha AL, Telles BA, et al. A 20-week program of resistance or concurrent exercise improves symptoms of schizophrenia: results of a blind, randomized controlled trial. Revista Brasileira de Psiquiatria. 2015;37(4):271-9.

23. Maurus I, Mantel C, Keller-Varady K, Schmitt A, Lembeck M, Röh A, et al. Resistance training in patients with schizophrenia: Concept and proof of principle trial. Journal of Psychiatric Research. 2020;120:72-82.

24. Korman N, Fox H, Skinner T, Dodd C, Suetani S, Chapman J, et al. Feasibility and Acceptability of a Student-Led Lifestyle (Diet and Exercise) Intervention Within a Residential Rehabilitation Setting for People With Severe Mental Illness, GO HEART (Group Occupation, Health, Exercise And Rehabilitation Treatment). Frontiers in Psychiatry. 2020;11.

25. Subramaniapillai M, Arbour-Nicitopoulos K, Duncan M, McIntyre RS, Mansur RB, Remington G, et al. Physical activity preferences of individuals diagnosed with schizophrenia or bipolar disorder. BMC Research Notes. 2016;9(1).

26. Strassnig MT, Signorile JF, Potiaumpai M, Romero MA, Gonzalez C, Czaja S, et al. High velocity circuit resistance training improves cognition, psychiatric symptoms and neuromuscular performance in overweight outpatients with severe mental illness. Psychiatry research. 2015;229(1-2):295-301.

27. Ashdown-Franks G, Firth J, Carney R, Carvalho AF, Hallgren M, Koyanagi A, et al. Exercise as Medicine for Mental and Substance Use Disorders: A Meta-review of the Benefits for Neuropsychiatric and Cognitive Outcomes. Sports medicine (Auckland, NZ). 2019.

28. Bredin SSD, Warburton DER, Lang DJ. The health benefits and challenges of exercise training in persons living with schizophrenia: a pilot study. Brain sciences. 2013;3(2):821-48.

29. Woodward M, Honer W, Su W, White R, Leonova O, Warburton D, et al. 160. Comparison of Frontal Cortical Volumes With Physical Fitness in Chronic Schizophrenia Patients Following Aerobic and Resistance Training Programs. Schizophrenia Bulletin. 2017;43(suppl1):S82-S.

30. State Of Queensland (Queensland Health). Community Care Unit Model of Service. Mental Health Alcohol and Other Drugs Branch DoH, editor. Fortitude Valley BC, QLD 4006: State of Queensland (Queensland Health)

; 2015. 30 p.

31. Meehan T, Stedman T, Parker S, Curtis B, Jones D. Comparing clinical and demographic characteristics of people with mental illness in hospital- and community-based residential rehabilitation units in Queensland. Australian Health Review. 2017;41(2):139-43.

32. Health). SoQQ. Community Care Unit Model of Service. Fortitude Valley BC, QLD, DoH MHAaODB; 2015.

33. Daumit GL, Dickerson FB, Wang N-Y, Dalcin A, Jerome GJ, Anderson CAM, et al. A Behavioral Weight-Loss Intervention in Persons with Serious Mental Illness. The New England Journal of Medicine. 2013;368(17):1594-602.

34. Hert M, Correll CU, Bobes J, Cetkovich‐Bakmas M, Cohen D, Asai I, et al. Physical illness in patients with severe mental disorders. I. Prevalence, impact of medications and disparities in health care. World Psychiatry. 2011;10(1):52-77.

35. Brown WJ, Bauman AE, Bull FC, Burton NW. Development of Evidence-based Physical Activity Recommendations for Adults (18-64 years). Government report. Department of Health and Ageing; 2012 Ausgust 2012.

36. Organization. WH. Global Recommendations on Physical Activity for Health. Switzerland: World Health Organization; 2010.

37. Brady D. 1-Rep Max (1RM) Calculator December 18, 2012 [Available from: https://www.sportsscience.co/tools/1-rep-max-1rm-calculator/.

38. Vancampfort D, Rosenbaum S, Probst M, Soundy A, Mitchell AJ, De Hert M, et al. Promotion of cardiorespiratory fitness in schizophrenia: a clinical overview and meta‐analysis. Acta Psychiatrica Scandinavica. 2015;132(2):131-43.

39. Andreasen NC, Pressler M, Nopoulos P, Miller D, Ho B-C. Antipsychotic Dose Equivalents and Dose-Years: A Standardized Method for Comparing Exposure to Different Drugs. Biological Psychiatry. 2010;67(3):255-62.

40. Firth J, Rosenbaum S, Stubbs B, Gorczynski P, Yung AR, Vancampfort D. Motivating factors and barriers towards exercise in severe mental illness: a systematic review and meta-analysis. 2016;46(14):2869-81.

41. Firth J, Rosenbaum S, Stubbs B, Vancampfort D, Carney R, Yung AR. Preferences and motivations for exercise in early psychosis. Acta Psychiatrica Scandinavica. 2016;134(1):83-4.

42. Ussher M, Stanbury L, Cheeseman V, Faulkner G. Physical Activity Preferences and Perceived Barriers to Activity Among Persons With Severe Mental Illness in the United Kingdom. Psychiatric Services. 2007;58(3):405-8.

43. Rosenbaum S, Morell R, Abdel-Baki A, Ahmadpanah M, Anilkumar T, Baie L, et al. Assessing physical activity in people with mental illness: 23-country reliability and validity of the simple physical activity questionnaire (SIMPAQ). BMC Psychiatry. 2020;20(1):1-12.

44. Chapman JJ, Fraser SJ, Brown WJ, Burton NW. The feasibility and acceptability of questionnaires and accelerometry for measuring physical activity and sedentary behaviour in adults with mental illness. Journal of Mental Health. 2015;24(5):299-304.

45. Suetani S, Chapman J, Korman N, Chapman C, Dodd C, Dark F, et al. A comparison study of three physical activity measurement tools examining acceptability in people with psychosis. Australasian psychiatry : bulletin of Royal Australian and New Zealand College of Psychiatrists. 2019:1039856219881957.

46. Bergman P. The number of repeated observations needed to estimate the habitual physical activity of an individual to a given level of precision.(Research Article)(Report). PLoS ONE. 2018;13(2):e0192117.

47. Williams J, Stubbs B, Richardson S, Flower C, Barr-Hamilton L, Grey B, et al. 'Walk this way': results from a pilot randomised controlled trial of a health coaching intervention to reduce sedentary behaviour and increase physical activity in people with serious mental illness.(Report). BMC Psychiatry. 2019;19(1).

48. Esliger DW, Rowlands AV, Hurst TL, Catt M, Murray P, Eston RG. Validation of the GENEA accelerometer. 2011;43(6).

49. Korman NH, Shah S, Suetani S, Kendall K, Rosenbaum S, Dark F, et al. Evaluating the Feasibility of a Pilot Exercise Intervention Implemented Within a Residential Rehabilitation Unit for People With Severe Mental Illness: GO HEART: (Group Occupational Health Exercise and Rehabilitation Treatment).(Report). Frontiers in Psychiatry. 2018;9.

50. Organisation. WH. WHO Disability Assessment Schedule 2.0 (WHODAS 2.0): World Health Organisation; 14 June 2018 [Available from: https://www.who.int/classifications/icf/more_whodas/en/.

51. Crippa JAS, Sanches RF, Hallak JEC, Loureiro SR, Zuardi AW. A structured interview guide increases Brief Psychiatric Rating Scale reliability in raters with low clinical experience. Acta Psychiatrica Scandinavica. 2001;103(6):465-70.

52. Ligon J, Thyer BA. Interrater reliability of the brief psychiatric rating scale used at a community‐based inpatient crisis stabilization unit. Journal of Clinical Psychology. 2000;56(4):583-7.

53. Lyne J, Renwick L, Grant T, Kinsella A, McCarthy P, Malone K, et al. Scale for the Assessment of Negative Symptoms structure in first episode psychosis. Psychiatry research. 2013;210(3):1191-7.

54. Schoemaker J, R G, M S. Interview-Guide-for-IG-SANS. Washington D.C

.

55. Alonso J, Ciudad A, Casado A, Gilaberte I. Measuring Schizophrenia Remission in Clinical Practice. The Canadian Journal of Psychiatry. 2008;53(3):202-6.

56. Wilson PM, Rodgers WM, Loitz CC, Scime G. “It's Who I Am… Really!’The Importance of Integrated Regulation in Exercise Contexts. J Appl Biobehav Res. 2006;11(2):79-104.

57. Bernard P, Romain AJ, Vancampfort D, Baillot A, Esseul E, Ninot G. Six minutes walk test for individuals with schizophrenia. Disability &amp; Rehabilitation, 2015, Vol37(11), p921-927. 2015;37(11):921-7.

58. ATS statement: guidelines for the six- minute walk test. American journal of respiratory and critical care medicine. 2002;166(1):111.

59. Jones CJ, Rikli RE, Beam WC. A 30-s Chair-Stand Test as a Measure of Lower Body Strength in Community-Residing Older Adults. Research Quarterly for Exercise and Sport. 1999;70(2):113-9.

60. Roberts HC, Denison HJ, Martin HJ, Patel HP, Syddall H, Cooper C, et al. A review of the measurement of grip strength in clinical and epidemiological studies: towards a standardised approach. Age and Ageing. 2011;40(4):423-9.

61. Blanchard CM, Rodgers WM, Spence JC, Courneya KS. Feeling state responses to acute exercise of high and low intensity. Journal of Science and Medicine in Sport. 2001;4(1):30-8.

62. McAuley E, Courneya KS. The Subjective Exercise Experiences Scale (SEES): development and preliminary validation. Journal of Sport & Exercise Psychology. 1994;16(2):163.

63. Leon AC, Davis LL, Kraemer HC. The role and interpretation of pilot studies in clinical research. Journal of Psychiatric Research. 2011;45(5):626-9.

References

1. Crippa JAS, Sanches RF, Hallak JEC, Loureiro SR, Zuardi AW. A structured interview guide increases Brief Psychiatric Rating Scale reliability in raters with low clinical experience. Acta Psychiatrica Scandinavica. 2001;103(6):465-70.

2. Ligon J, Thyer BA. Interrater reliability of the brief psychiatric rating scale used at a community‐based inpatient crisis stabilization unit. Journal of Clinical Psychology. 2000;56(4):583-7.

3. Bernard P, Romain AJ, Vancampfort D, Baillot A, Esseul E, Ninot G. Six minutes walk test for individuals with schizophrenia. Disability &amp; Rehabilitation, 2015, Vol37(11), p921-927. 2015;37(11):921-7.

4. ATS statement: guidelines for the six- minute walk test. American journal of respiratory and critical care medicine. 2002;166(1):111.

5. Herron LR, Casey CJ, Tolusso VD, Casey LK, Bishop AP. Influence Of Short-term Training On Performance And Reliability Of A 1-minute Push-up Test: 3265 Board #330 June 3, 3: 30 PM - 5: 00 PM. Medicine & Science in Sports & Exercise. 2016;48(5S Suppl 1):935-6.

6. Braun V, Clarke V. Using thematic analysis in psychology. Qualitative Research in Psychology. 2006;3(2):77-101.

7. Maurus I, Mantel C, Keller-Varady K, Schmitt A, Lembeck M, Röh A, et al. Resistance training in patients with schizophrenia: Concept and proof of principle trial. Journal of Psychiatric Research. 2020;120:72-82.

8. Stubbs B, Vancampfort D, Hallgren M, Firth J, Veronese N, Solmi M, et al. EPA guidance on physical activity as a treatment for severe mental illness: a meta-review of the evidence and Position Statement from the European Psychiatric Association (EPA), supported by the International Organization of Physical Therapists in Mental Health (IOPTMH). European Psychiatry. 2018;54:124-44.

9. Brown WJ, Bauman AE, Bull FC, Burton NW. Development of Evidence-based Physical Activity Recommendations for Adults (18-64 years). Government report. Department of Health and Ageing; 2012 Ausgust 2012.

10. Organization. WH. Global Recommendations on Physical Activity for Health. Switzerland: World Health Organization; 2010.

11. Brady D. 1-Rep Max (1RM) Calculator December 18, 2012 [Available from: <https://www.sportsscience.co/tools/1-rep-max-1rm-calculator/>.

12. Vancampfort D, Rosenbaum S, Probst M, Soundy A, Mitchell AJ, De Hert M, et al. Promotion of cardiorespiratory fitness in schizophrenia: a clinical overview and meta‐analysis. Acta Psychiatrica Scandinavica. 2015;132(2):131-43.

13. Williams J, Stubbs B, Richardson S, Flower C, Barr-Hamilton L, Grey B, et al. 'Walk this way': results from a pilot randomised controlled trial of a health coaching intervention to reduce sedentary behaviour and increase physical activity in people with serious mental illness.(Report). BMC Psychiatry. 2019;19(1).

# **Appendix 3a.** Intervention structure.

|  |  | **Resistance Training (RT)** | **Aerobic Interval Training (AIT)** |
| --- | --- | --- | --- |
|  | Warm up | 5 minutes aerobic RPE (1-2), + 5 minutes stretching | 10 minutes aerobic (RPE 1-2) |
|  | Equipment | A variety of standard resistance training equipment (hand weights, kettle bells, resistance bands, squat rack, chest press bar and floor mats appropriate for small clinical gym settings | Treadmill, elliptical, rowing machine, seated bicycle |
|  | Prescription | 6-8 main muscle groups, arms, shoulders, back, core, lower limbs  Upper and lower body exercises were alternated between sessions to facilitate recovery.  3-4 sets of 8-12 repetitions, ~ 90 sec passive rest interval between sets until active exercise session duration reached  The content and weights of each session was recorded using standard protocols | Any aerobic activity that aims to increase cardiorespiratory fitness using preferred gym equipment and an interval training structure  4 minutes active aerobic exercise, alternating with 3 minutes active recovery (reduced intensity of the active recovery interval, RPE 1-2) until session duration reached  The equipment and active intervals were recorded each session using standard protocols |
|  | Cool down | 5 minutes stretch | 5 minutes stretch |

# **Appendix 3b.** Training progression.

| **Time point** | **RPE** | **Duration of active exercise** | **Prescription** |
| --- | --- | --- | --- |
| Session 1 | 2-3  (RT – 40% 1-RM) | 30 mins | Familiarisation with equipment and correct exercise technique. |
| Week 1-3 | 2-3 | 30 mins | Prescription reviewed weekly: progression individualised and could be regressed/progressed per capacity and exercise history. RT progression through increases in load.  AIT progression through increases in intensity. |
| Week 4 | 4  (RT – 40-70%) | 40 mins | Progression individualised and could be regressed/progressed per response |
| Week 5-8 | ≥4 | 45-55 mins | Progression individualised and could be regressed/progressed per response |

Abbreviations: RPE = rate of perceived exertion; RM = repetition max; RT – resistance training; AIT – aerobic interval training, mins – minutes.

# **Appendix 4.** Proforma CERT checklist

| **Section** | **Item number** | **Description** | **Details** | **Location (page)** |
| --- | --- | --- | --- | --- |
| WHAT:  materials | 1 | Detailed description of the type of exercise equipment | RT - hand weights, kettle bells, resistance bands and a chest press bar, squat rack and floor mat appropriate for small clinical gym settings to enable replication of the study without substantial investment in specialised equipment or gym spaces. AIT - equipment included a stationary bike, treadmill, rowing ergometer and elliptical machine. | Materials and Methods: Resistance Training Intervention and Aerobic Interval Training, page 7-8 |
| WHO:  provider | 2 | Detailed description of the qualifications, teaching/supervising expertise, and/or training  undertaken by the exercise instructor | Fully supervised by an accredited exercise physiologist (AEP), with experience in mental health settings, and in delivering exercise to untrained and vulnerable health populations  Training for AEP’s was conducted in the specific delivery of the intervention such that participant programs were be standardised for each condition | Material and Methods: Interventional Exercise(s), page 5  Material and Methods: Fidelity, page 8 |
| HOW:  delivery | 3 | Describe whether exercises are performed individually or in a group | Participants choose whether to engage in individual sessions or in small groups.  During COVID 19 pandemic all participants were only offered individual sessions. | Material and Methods: Strategies to enhance endurance, page 6  Results: Real world trial considerations, page 14 |
|  | 4 | Describe whether exercises are supervised or unsupervised and how they are delivered | All intervention sessions fully supervised by an accredited exercise physiologist (AEP) | Material and Methods: Interventional Exercise(s), page 5 |
|  | 5 | Detailed description of how adherence to exercise is measured and reported | Participation in an individual session was recorded if greater than 50% of the session was attended. Individual participation in ≥ 65% of the 24 available sessions (16 /24 sessions) was considered feasible. Feasibility was considered if greater than 70% of participants allocated per condition completed the weight week intervention | Material and Methods: Primary outcomes, page 9-10 |
|  | 6 | Detailed description of motivation strategies | 15-minute fortnightly health coaching session focusing on self-monitoring, goal setting, and building self-efficacy for exercise. Participants were encouraged to play music of their choice to enhance pleasure and could choose whether to engage in individual sessions or in small groups. The intervention was based on the capability, opportunity and motivation (COM-B) model of behaviour change (Michie et al., 2011)and theoretical domains of the COM-B model have been articulated by the Theoretical Domains Framework (TDF) used in health care interventions (Atkins et al., 2017). | Material and methods: Strategies to enhance endurance, page 6 |
|  | 7a | Detailed description of the decision rule(s) for determining exercise progression | Both conditions were aimed at moderate intensity exercise that was balanced for total number of sessions and volume. Active exercise duration started as 30-minutes duration. Volume was progressed to 40 minutes per session by week four of the intervention, with total duration of the session approximately 55 minutes, including warm up and cool down.Exercise prescription was reviewed weekly by the AEP and exercise progressed/regressed according to individual response. |  |
|  | 7b | Detailed description of how the exercise program was progressed | Intensity of the intervention was monitored using the Borg Category Ratio 10 scale of Perceived Exertion scale (RPE), (Heath, 1998)- a 10 point scale to assess subjective perception of effort that has been calibrated against exercise intensity and will guide intensity of exercise prescription. The aim was a starting RPE of 2-3 in week 1, with progression to RPE 4 by week 3. |  |
|  | 8 | Detailed description of each exercise to enable replication (e.g. photographs, illustrations ,  video etc) | The content and weights of each session was recorded using standard protocols. | Supplementary material X; example of an RT and and AIT program  Supplementary material X: photos of each session |
|  | 9 | Detailed description of any home program component (e.g. other exercises, stretching etc) | No specific home program was prescribed, however participants could engage in the same program developed for the intervention in their own time, but there were study restrictions around engaging in other exercise outside of the randomised condition to prevent contamination of results. | Materials and Methods:  Randomisation, page 4 |
|  | 10 | Describe whether there are any non-exercise components (e.g. education, cognitive  behavioural therapy, massage etc) | 15-minute fortnightly health coaching session focusing on self-monitoring, goal setting, and building self-efficacy for exercise. | Material and methods: Strategies to enhance endurance, page 6 |
|  | 11 | Describe the type and number of adverse events that occurred during exercise | Adverse events (AE’s) were monitored closely during the intervention using an AE protocol  There were 3 serious adverse events, none related to the exercise intervention with no difference between groups. There were 39 minor adverse events related to exercise; 34 in the RT group versus 5 in the AIT group, with the majority of RT-related AE’s being muscle soreness (n=22) or joint stiffness (n=7). The RT group had a significantly higher number of AE’s per participant compared to AIT, z= 3.23, p=0.01, see Figure 2. There were no drop- outs due to exercise -related AE’s in either group | Material and methods: primary outcomes, Adverse events, page 9  Material and methods: Primary outcomes, page 13 and Table 2a |
| WHERE:  location | 12 | Describe the setting in which the exercises are performed | The study was conducted within the shared courtyard, using pre-existing gym facilities and equipment maintained by each residential rehabilitation unit. | Material and Methods: Interventional Exercise(s), page 5 |
| WHEN, HOW MUCH:  dosage | 13 | Detailed description of the exercise intervention including, but not limited to, number of  exercise repetitions/sets/sessions, session duration, intervention/program duration etc | Sessions involved a warm-up (AIT - 10-minute aerobic warm up; RT - 5-minute aerobic & 5-minute stretching warm up), active intervention and 5-minute cool down phase. Active exercise duration started as 30-minutes duration. Volume was progressed to 40 minutes per session by week four of the intervention, with total duration of the session approximately 55 minutes, including warm up and cool down.  RT - to occur on 2 or more days per week, involving main muscle groups. An initial assessment of muscle strength was assessed using the 12 repetition maximum test (12-RM) for relevant large muscle groups (the squat and bench press) to guide exercise prescription. Participants completed three to four sets of 8-12 repetitions to moderate fatigue (RPE 3-4/10), and approximately 90 seconds passive rest interval between sets. Week 1 started with 40% of 1-RM. As strength increased, the RT was progressed using heavier weights, as per capacity of the individual and in accordance with their RPE. Approximately six to eight exercises across main muscle groups used in activities of daily living were prescribed per session until a total of 30 minutes of active exercise was completed.  The AEP will aim for the participant to maintain an RPE at 2–3 for the first 2 weeks, then increased to a minimum RPE of 4/10 by week 3 and further increased each week as per individual participant's capacity. Following warm-up, participants will begin interval sets, beginning with four minutes of moderate intensity exercise (RPE 3-4) interspersed with three minutes of active recovery (reduced intensity interval, RPE 1- 2) until a total of 30 minutes of AIT is completed. Volume will be progressed to 40 minutes by week 4. | Material and Methods: Interventional Exercise(s), page 5  Material and Methods: Resistance Training, page 6  Material and Methods: Aerobic Interval training, page 7 |
| TAILORING:  What, how | 14a | Describe whether the exercises are generic (one size fits all) or tailored whether tailored to  the individual | Exercise prescription was reviewed weekly by the AEP and exercise progressed/regressed according to individual response. | Material and Methods: Interventional Exercise(s), page 6 |
|  | 14b | Detailed description of how exercises are tailored to the individual | Equipment was chosen as the resistance and speed could be modified to suit individual participant capacity and preference. | Material and Methods: Aerobic Interval training, page 8 |
|  | 15 | Describe the decision rule for determining the starting level at which people commence an  exercise program (such as beginner, intermediate, advanced etc) | The aim was a starting RPE of 2-3 in week 1, with progression to RPE 4 by week 3.  RT - An initial assessment of muscle strength was assessed using the 12 repetition maximum test (12-RM) for relevant large muscle groups (the squat and bench press) to guide exercise prescription.  AIT - Initial prescription was aimed at an intensity achievable for participants with low initial CRF but progressed to reach moderate intensity by week 3 | Material and Methods: Interventional Exercise(s), page 5  Material and Methods: Resistance Training, page 7  Material and Methods: Aerobic Interval Training, page 8 |
| HOW WELL:  planned, actual | 16a | Describe how adherence or fidelity to the exercise intervention is assessed/measured | Programs were reviewed against protocol criteria at intervals by the research team to ensure prescriptions were conducted within the scope of the intervention. | Material and Methods: Fidelity, page 8 |
|  | 16b | Describe the extent to which the intervention was delivered as planned | Completion of the entire eight week intervention rates were 24/27 (88.8%) in each condition, with no difference between the groups. Median participation rates were 23.5 (IQR 4) out of a total of 24 sessions, and no difference between groups. | Results: Primary outcomes, page 13 |

Abbreviations: RT – resistance training, AIT – aerobic interval training

**References**

Atkins, L., Francis, J., Islam, R., O'Connor, D., Patey, A., Ivers, N., Foy, R., Duncan, E. M., Colquhoun, H., Grimshaw, J. M., Lawton, R., & Michie, S., 2017. A guide to using the Theoretical Domains Framework of behaviour change to investigate implementation problems. Implement Sci, 12(1), 77-77. doi:10.1186/s13012-017-0605-9

Heath, E. M., 1998. Borg's Perceived Exertion and Pain Scales. Medicine& Science in Sports & Exercise, 30(9), 1461. doi:10.1249/00005768-199809000-00018

Michie, S., van Stralen, M. M., & West, R., 2011. The behaviour change wheel: A new method for characterising and designing behaviour change interventions.(Research). Implementation Science, 6, 42

# **Appendix 5.** RPE data for week 3 and week 8.

|  |  | Week 3 | |  | Week 8 | |  |
| --- | --- | --- | --- | --- | --- | --- | --- |
|  | **Exercise group** | **No. participants** | **RPE median, (IQR)** | **Mann Whitney-U,**  **p value** | **No. participants** | **RPE median, (IQR)** | **Mann Whitney-U,**  **p value** |
|  | Resistance Training | n=18 | 3.1 (1) | 287.5 (p=0.06) | n=16 | 3.9 (1.8) | 219.5  (p=0.2) |
|  | Aerobic Training | n=24 | 3 (0.78) |  | n=22 | 3.3 (0.81) |  |

No. = number, RPE – rate of perceived exertion, IQR – interquartile range,

# **Appendix 6**. Associations between baseline variables and dependent variables

| Independent variables | | Positive Well Being | | Psychological Distress | | Fatigue | |
| --- | --- | --- | --- | --- | --- | --- | --- |
|  |  | Week 3 | Week 8 | Week 3 | Week 8 | Week 3 | Week 8 |
| Demographics | Age | 0.12 (0.409) | 0.15 (0.321) | 0.19 (0.173) | -0.01 (0.953) | 0.09 (0.537) | 0.07 (0.638) |
|  | Gender | 0.01 (0.968) | -0.03 (0.828) | 0.00 (0.992) | 0.11 (0.447) | 0.11 (0.423) | 0.11 (0.476) |
|  | BMI | 0.19 (0.184) | -0.15 (0.316) | -0.06 (0.692) | -0.07 (0.651) | 0.17 (0.221) | 0.17 (0.246) |
|  | Duration of rehabilitation | -0.10 (0.470) | -0.25 (0.091) | -0.23 (0.106) | -0.43 (0.002) | 0.08 (0.571) | 0.01 (0.950) |
|  | Primary diagnosis | 7.40 (0.286) | 7.12 (0.310) | 5.51 (0.480) | 5.78 (0.448) | 11.87 (0.065) | 4.16 (0.655) |
| BREQ-3 | Amotivation | -0.07 (0.618) | 0.21 (0.161) | -0.34 (0.014)* | -0.15 (0.320) | -0.01 (0.932) | 0.03 (0.841) |
|  | External regulation | 0.22 (0.117) | -0.12 (0.416) | -0.11 (0.449) | -0.11 (0.468) | 0.25 (0.068) | 0.24 (0.104) |
|  | Introjected regulation | 0.23 (0.105) | 0.18 (0.216) | 0.04 (0.765) | 0.10 (0.478) | 0.02 (0.866) | 0.16 (0.275) |
|  | Integrated regulation | 0.12 (0.386) | 0.12 (0.430) | -0.16 (0.261) | -0.18 (0.216) | 0.07 (0.632) | 0.29 (0.045) |
|  | Intrinsic regulation | 0.17 (0.224) | 0.17 (0.260) | -0.11 (0.442) | 0.06 (0.673) | 0.11 (0.441) | -0.02 (0.918) |
|  | Identified regulation | 0.08 (0.552) | 0.04 (0.777) | -0.14 (0.336) | -0.11 (0.441) | 0.22 (0.123) | 0.06 (0.710) |
|  | Autonomous regulation | 0.17 (0.233) | 0.14 (0.351) | -0.15 (0.285) | -0.08 (0.608) | 0.14 (0.341) | 0.15 (0.312) |
|  | Controlled regulation | 0.29 (0.039)* | 0.01 (0.953) | -0.03 (0.849) | -0.04 (0.813) | 0.17 (0.219) | 0.22 (0.139) |
|  | RAI | 0.01 (0.957) | 0.07 (0.645) | 0.00 (0.996) | -0.05 (0.727) | 0.00 (0.998) | 0.02 (0.904) |
| Symptoms | Total BPRS | 0.16 (0.272) | 0.10 (0.506) | 0.16 (0.269) | -0.06 (0.692) | -0.06 (0.698) | 0.07 (0.614) |
|  | Mood at baseline | 0.29 (0.037)* | -0.09 (0.563) | 0.18 (0.194) | -0.02 (0.871) | -0.12 (0.392) | 0.01 (0.936) |
|  | Negative symptoms | 0.18 (0.199) | -0.24 (0.094) | 0.19 (0.171) | -0.11 (0.445) | 0.22 (0.123) | 0.18 (0.217) |
|  | Duration of illness | 0.36 (0.010)* | 0.04 (0.782) | 0.21 (0.126) | -0.05 (0.748) | -0.09 (0.547) | 0.04 (0.771) |
|  | Total functioning | 0.28 (0.048)* | -0.08 (0.603) | 0.17 (0.236) | 0.02 (0.906) | 0.05 (0.739) | 0.19 (0.190) |
| Medications | Physical health meds | 0.14 (0.311) | -0.10 (0.493) | 0.09 (0.503) | -0.12 (0.424) | 0.17 (0.225) | 0.19 (0.204) |
|  | Olanzapine equivalents | 0.00 (0.985) | -0.08 (0.571) | 0.06 (0.661) | 0.06 (0.695) | -0.25 (0.074) | 0.08 (0.584) |
|  | Psychiatric medications | -0.08 (0.582) | 0.01 (0.971) | 0.20 (0.146) | 0.19 (0.206) | -0.24 (0.092) | 0.11 (0.459) |
| Physical activity | Six-minute walking test | -0.13 (0.356) | 0.00 (0.999) | -0.01 (0.919) | 0.04 (0.805) | 0.03 (0.820) | -0.09 (0.537) |
|  | MVPA (subjective) | -0.33 (0.017)* | -0.03 (0.852) | -0.16 (0.247) | 0.02 (0.885) | 0.06 (0.680) | 0.07 (0.624) |
|  | Exercise group preference | 1.54 (0.463) | 0.64 (0.726) | 0.64 (0.726) | 1.09 (0.580) | 0.89 (0.640) | 1.31 (0.519) |
|  | Within intervention RPE | -0.05 (0.756) | -0.15 (0.374) | -0.05 (0.774) | -0.23 (0.158) | 0.26 (0.094) | 0.15 (0.374) |

BMI = Body Mass Index; BPRS = Brief Psychiatric Rating Scale; BREQ= Behavioural Regulation in Exercise Questionnaire; MVPA = moderate/vigorous physical activity

# **Appendix 7.** Mixed Model Repeated Measures analysis

| SEES domain | Variable | β (SE) | *p*-value |
| --- | --- | --- | --- |
| Positive wellbeing | Timepoint (post-pre) | 2.92 (0.73) | <0.001* |
|  | Intervention group | -0.31 (1.19) | 0.79 |
|  | Week (week 3 vs week 8) | 1.28 (0.75) | 0.09 |
|  | Age | -0.11 (0.06) | 0.04* |
|  | Sex | 1.83 (1.11) | 0.10 |
|  | BMI | 0.11 (0.08) | 0.14 |
|  | Self-report MVPA | 0.01 (0.00) | 0.11 |
|  | Mood | -0.10 (0.37) | 0.78 |
|  | Olanzapine equivalents | 0.05 (0.03) | 0.07 |
|  | Duration of illness | 0.03 (0.08) | 0.68 |
|  | Amotivation | -0.36 (0.86) | 0.68 |
|  | Controlled regulation | 0.37 (0.63) | 0.56 |
|  | Timepoint (post-pre) * intervention group | 0.12 (1.03) | 0.91 |
|  | Timepoint (post-pre) * Week (week 3 vs week 8) | 1.03 (1.05) | 0.32 |
|  | Intervention group * Week (week 3 vs week 8) | -1.57 (1.06) | 0.14 |
|  | Timepoint (post-pre) * intervention group * Week (week 3 vs week 8) | -0.57 (1.48) | 0.70 |
| Psychological  distress | Timepoint (post-pre) | 2.41 (0.74) | 0.001* |
|  | Intervention group | 1.12 (1.10) | 0.31 |
|  | Week (week 3 vs week 8) | 0.94 (0.76) | 0.22 |
|  | Age | 0.05 (0.05) | 0.35 |
|  | Sex | 0.54 (0.99) | 0.59 |
|  | BMI | -0.05 (0.07) | 0.48 |
|  | Self-report MVPA | 0.00 (0.00) | 0.51 |
|  | Depressed mood | 0.28 (0.32) | 0.40 |
|  | Olanzapine equivalents | -0.02 (0.02) | 0.39 |
|  | Duration of illness | 0.00 (0.07) | 0.95 |
|  | Amotivation | -0.36 (0.77) | 0.64 |
|  | Controlled regulation | 0.75 (0.56) | 0.19 |
|  | Timepoint (post-pre) * intervention group | -0.17 (1.04) | 0.87 |
|  | Timepoint (post-pre) * Week (week 3 vs week 8) | -0.78 (1.06) | 0.46 |
|  | Intervention group * Week (week 3 vs week 8) | -1.01 (1.07) | 0.35 |
|  | Timepoint (post-pre) * intervention group * Week (week 3 vs week 8) | 1.11 (1.50) | 0.46 |
| Fatigue | Timepoint (post-pre) | -1.92 (1.05) | 0.06 |
|  | Intervention group | 3.02 (1.33) | 0.02* |
|  | Week (week 3 vs week 8) | -0.12 (1.07) | 0.91 |
|  | Age | -0.04 (0.05) | 0.42 |
|  | Sex | 1.10 (1.07) | 0.31 |
|  | BMI | -0.06 (0.07) | 0.433 |
|  | Self-report MVPA | 0.01 (0.00) | 0.157 |
|  | Depressed mood | 0.80 (0.35) | 0.028* |
|  | Olanzapine equivalents | 0.01 (0.03) | 0.670 |
|  | Duration of illness | -0.03 (0.08) | 0.750 |
|  | Amotivation | 0.39 (0.84) | 0.646 |
|  | Controlled regulation | 1.72 (0.61) | 0.007* |
|  | Timepoint (post-pre) * intervention group | 3.11 (1.48) | 0.04* |
|  | Timepoint (post-pre) * Week (week 3 vs week 8) | -0.54 (1.51) | 0.72 |
|  | Intervention group * Week (week 3 vs week 8) | -0.09 (1.52) | 0.95 |
|  | Timepoint (post-pre) * intervention group * Week (week 3 vs week 8) | 1.43 (2.14) | 0.50 |

*significant difference p value <0.05, BMI – Body mass Index, SEES – Subjective Exercise Experiences Scale

# **Appendix 8.** Table of outcome measures (LS means) week three and week eight.

|  |  | **Week 3** | | | **Week 8** | | |
| --- | --- | --- | --- | --- | --- | --- | --- |
|  |  | **LS mean (pre) SE** | **LS mean**  **(post) SE** | **Within group effect size (Hedge’s g)** | **LS mean (pre)nSE** | **LS mean**  **(post) SE** | **Within group effect size (Hedge’s g)** |
|  | **Positive well being** |  | | | | | |
|  | Resistance Training | 16.2 (1.1) | 19.5 (1.1) | 0.77 | 15.8 (1.1) | 18.6 (1.1) | 0.71 |
|  | Aerobic Training | 17.2 (0.8) | 20 (0.9) | 0.64 | 17.1 (0.9) | 21 (0.9) | 0.84 |
|  | **Psychological Distress** |  |  |  |  |  |  |
|  | Resistance Training | 8.8 (0.9) | 5.9 (0.9) | -0.68 | 7.6 (0.9) | 6.1 (0.9) | -0.57 |
|  | Aerobic Training | 7.7 (0.8) | 4.9 (0.8) | -0.56 | 9.3 (0.8) | 6.2 (0.8) | -0.75 |
|  | **Fatigue** |  | | | | | |
|  | Resistance Training | 11.6 (1.3) | 13 (1.3) | 0.25 | 9.5 (1.3) | 13.5 (1.4) | 0.39 |
|  | Aerobic Training | 11.3 (1.1) | 9.4 (1.1) | -0.35 | 11.3 (1.1) | 9 (1.1) | -0.49 |

LS – least squares mean, CI – confidence interval, SE – standard error

# **Appendix 9.** Figures for week three and week eight – Pre versus post (a) positive wellbeing scores, (b) psychological distress scores, and (c) fatigue scores across treatment group.


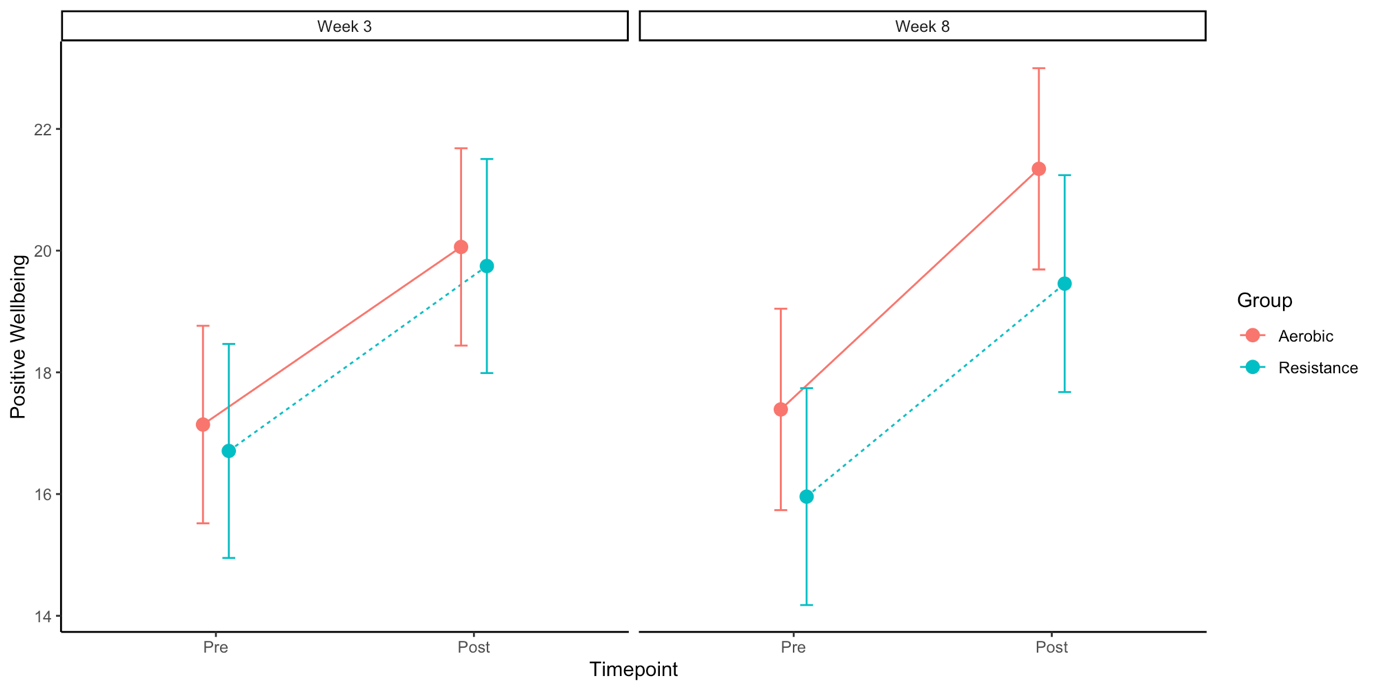

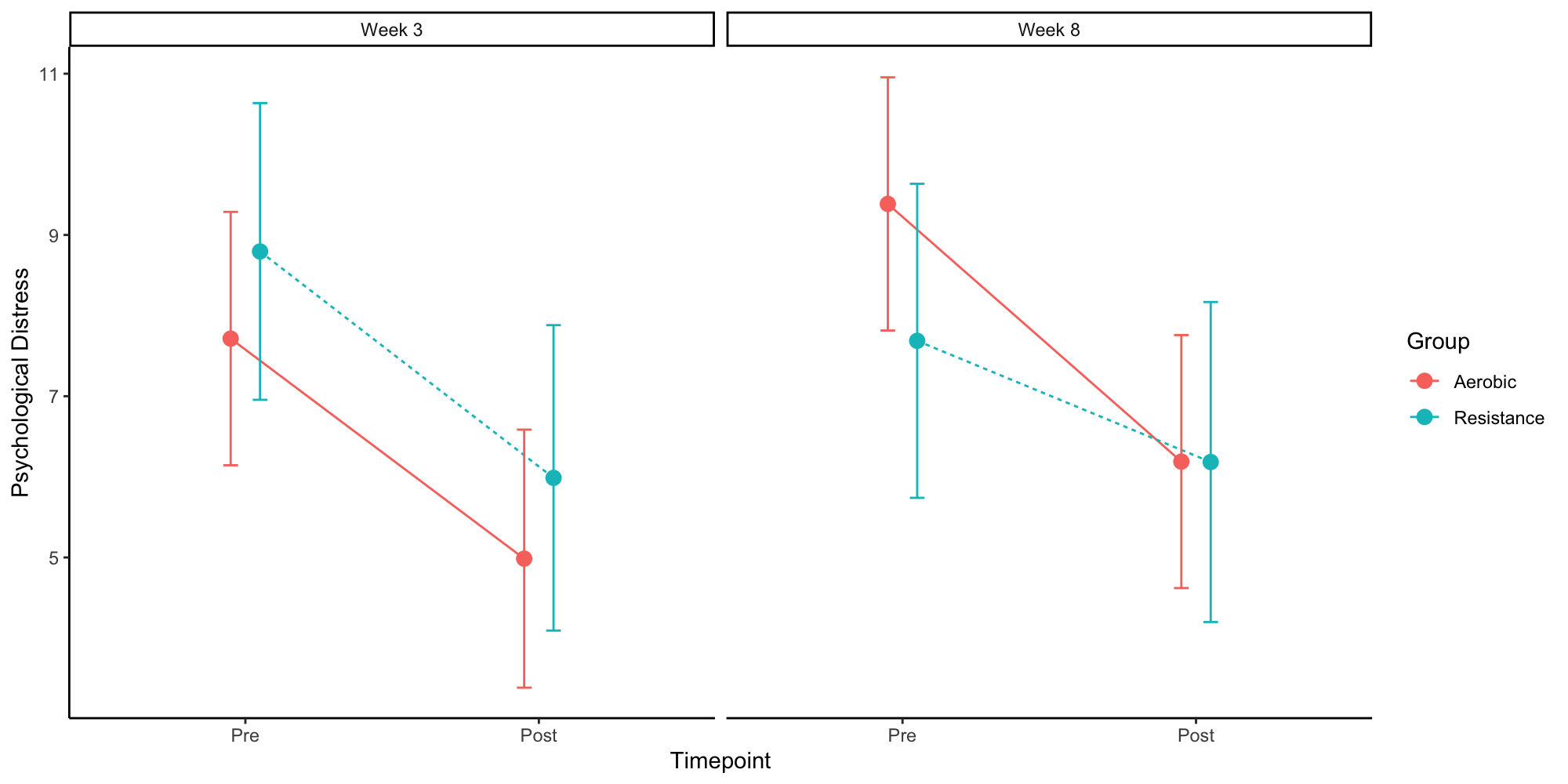

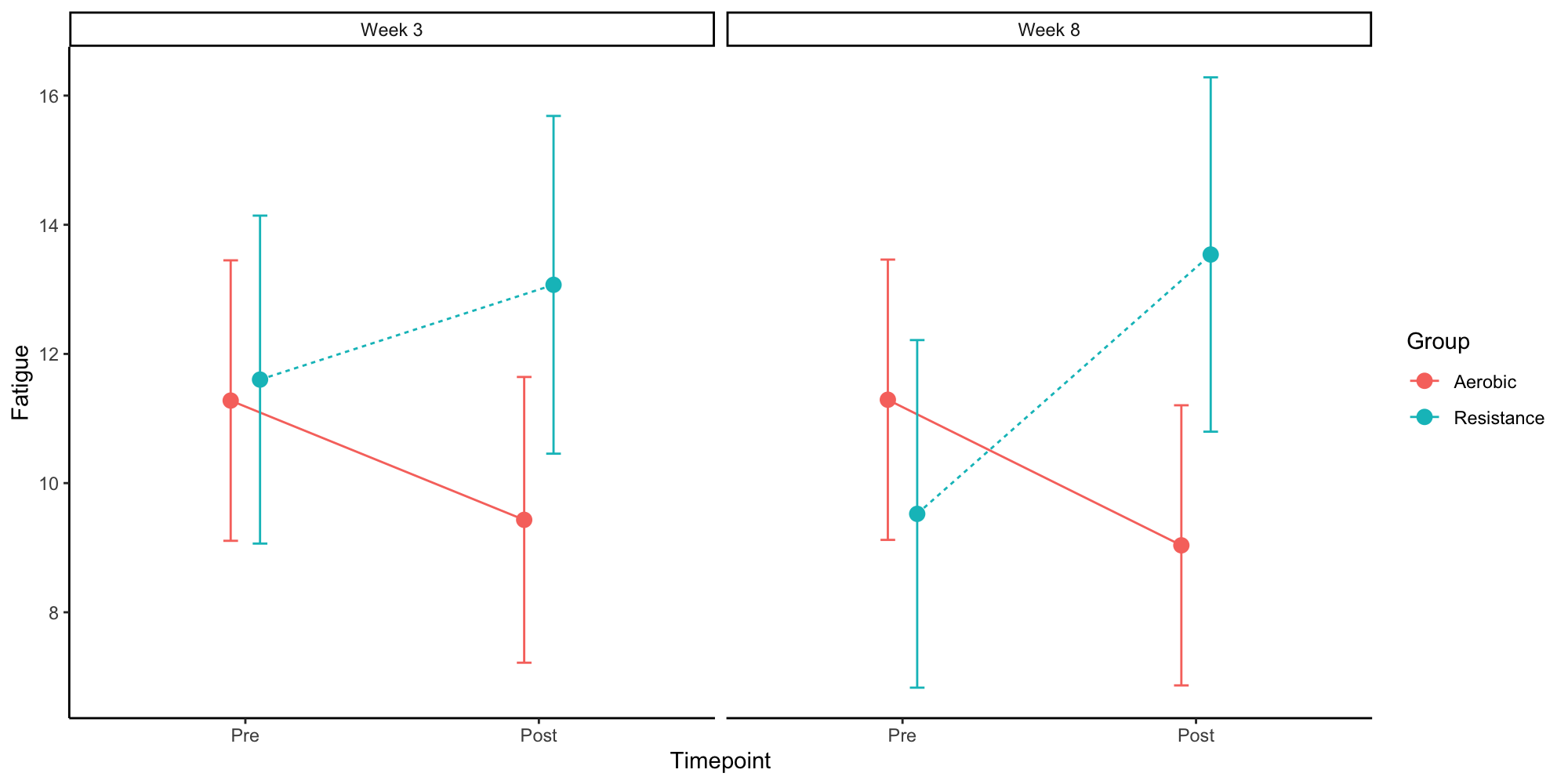


# **Appendix 10.** Multicollinearity model.

| **FATIGUE** | | | | |  |  | | | | | |
| --- | --- | --- | --- | --- | --- | --- | --- | --- | --- | --- | --- |
|  | |  | |  |  |  | | | | | |
| **GVIF** | | **Df** | | **GVIF^(1/(2*Df))** | **Interacts With** | **Other Covariates** | | | | | |
| 1.2251835 | | 7 | | 1.014612205 | newgroups, week | MVPA_SR_baseline, Age, gender, correctmood_baseline, BMI_baseline, olanzapineequivalents, Durationillness, amotivation_baseline, controlledregulation_baseline | | | | | |
| 1.2251835 | | 7 | | 1.014612205 | timepoint, week | MVPA_SR_baseline, Age, gender, correctmood_baseline, BMI_baseline, olanzapineequivalents, Durationillness, amotivation_baseline, controlledregulation_baseline | | | | | |
| 1.2251835 | | 7 | | 1.014612205 | timepoint, newgroups | MVPA_SR_baseline, Age, gender, correctmood_baseline, BMI_baseline, olanzapineequivalents, Durationillness, amotivation_baseline, controlledregulation_baseline | | | | | |
| 1.3878073 | | 1 | | 1.178052325 | -- | timepoint, newgroups, week, Age, gender, correctmood_baseline, BMI_baseline, olanzapineequivalents, Durationillness, amotivation_baseline, controlledregulation_baseline | | | | | |
| 1.6401837 | | 1 | | 1.280696573 | -- | timepoint, newgroups, week, MVPA_SR_baseline, gender, correctmood_baseline, BMI_baseline, olanzapineequivalents, Durationillness, amotivation_baseline, controlledregulation_baseline | | | | | |
| 1.1995247 | | 1 | | 1.095228171 | -- | timepoint, newgroups, week, MVPA_SR_baseline, Age, correctmood_baseline, BMI_baseline, olanzapineequivalents, Durationillness, amotivation_baseline, controlledregulation_baseline | | | | | |
| 1.5857091 | | 1 | | 1.25924942 | -- | timepoint, newgroups, week, MVPA_SR_baseline, Age, gender, BMI_baseline, olanzapineequivalents, Durationillness, amotivation_baseline, controlledregulation_baseline | | | | | |
| 1.6317201 | | 1 | | 1.277388012 | -- | timepoint, newgroups, week, MVPA_SR_baseline, Age, gender, correctmood_baseline, olanzapineequivalents, Durationillness, amotivation_baseline, controlledregulation_baseline | | | | | |
| 1.2809032 | | 1 | | 1.131769948 | -- | timepoint, newgroups, week, MVPA_SR_baseline, Age, gender, correctmood_baseline, BMI_baseline, Durationillness, amotivation_baseline, controlledregulation_baseline | | | | | |
| 2.3649979 | | 1 | | 1.537854963 | -- | timepoint, newgroups, week, MVPA_SR_baseline, Age, gender, correctmood_baseline, BMI_baseline, olanzapineequivalents, amotivation_baseline, controlledregulation_baseline | | | | | |
| 1.2105067 | | 1 | | 1.100230292 | -- | timepoint, newgroups, week, MVPA_SR_baseline, Age, gender, correctmood_baseline, BMI_baseline, olanzapineequivalents, Durationillness, controlledregulation_baseline | | | | | |
| 1.6974066 | | 1 | | 1.302845567 | -- | timepoint, newgroups, week, MVPA_SR_baseline, Age, gender, correctmood_baseline, BMI_baseline, olanzapineequivalents, Durationillness, amotivation_baseline | | | | | |
| **POSITIVE WELLBEING** | | | | |  |  | | | | | |
| **GVIF** | | **Df** | | **GVIF^(1/(2*Df))** | **Interacts With** | **Other Predictors** | | | | | |
| 1.225184 | | 7 | | 1.014612 | newgroups, week | MVPA_SR_baseline, Age, gender, correctmood_baseline, BMI_baseline, olanzapineequivalents, Durationillness, amotivation_baseline, controlledregulation_baseline | | | | | |
| 1.225184 | | 7 | | 1.014612 | timepoint, week | MVPA_SR_baseline, Age, gender, correctmood_baseline, BMI_baseline, olanzapineequivalents, Durationillness, amotivation_baseline, controlledregulation_baseline | | | | | |
| 1.225184 | | 7 | | 1.014612 | timepoint, newgroups | MVPA_SR_baseline, Age, gender, correctmood_baseline, BMI_baseline, olanzapineequivalents, Durationillness, amotivation_baseline, controlledregulation_baseline | | | | | |
| 1.387807 | | 1 | | 1.178052 | -- | timepoint, newgroups, week, Age, gender, correctmood_baseline, BMI_baseline, olanzapineequivalents, Durationillness, amotivation_baseline, controlledregulation_baseline | | | | | |
| 1.640184 | | 1 | | 1.280697 | -- | timepoint, newgroups, week, MVPA_SR_baseline, gender, correctmood_baseline, BMI_baseline, olanzapineequivalents, Durationillness, amotivation_baseline, controlledregulation_baseline | | | | | |
| 1.199525 | | 1 | | 1.095228 | -- | timepoint, newgroups, week, MVPA_SR_baseline, Age, correctmood_baseline, BMI_baseline, olanzapineequivalents, Durationillness, amotivation_baseline, controlledregulation_baseline | | | | | |
| 1.585709 | | 1 | | 1.259249 | -- | timepoint, newgroups, week, MVPA_SR_baseline, Age, gender, BMI_baseline, olanzapineequivalents, Durationillness, amotivation_baseline, controlledregulation_baseline | | | | | |
| 1.63172 | | 1 | | 1.277388 | -- | timepoint, newgroups, week, MVPA_SR_baseline, Age, gender, correctmood_baseline, olanzapineequivalents, Durationillness, amotivation_baseline, controlledregulation_baseline | | | | | |
| 1.280903 | | 1 | | 1.13177 | -- | timepoint, newgroups, week, MVPA_SR_baseline, Age, gender, correctmood_baseline, BMI_baseline, Durationillness, amotivation_baseline, controlledregulation_baseline | | | | | |
| 2.364998 | | 1 | | 1.537855 | -- | timepoint, newgroups, week, MVPA_SR_baseline, Age, gender, correctmood_baseline, BMI_baseline, olanzapineequivalents, amotivation_baseline, controlledregulation_baseline | | | | | |
| 1.210507 | | 1 | | 1.10023 | -- | timepoint, newgroups, week, MVPA_SR_baseline, Age, gender, correctmood_baseline, BMI_baseline, olanzapineequivalents, Durationillness, controlledregulation_baseline | | | | | |
| 1.697407 | | 1 | | 1.302846 | -- | timepoint, newgroups, week, MVPA_SR_baseline, Age, gender, correctmood_baseline, BMI_baseline, olanzapineequivalents, Durationillness, amotivation_baselin | | | | | |
| **PSYCHOLOGICAL DISTRESS** | | | | |  |  | | | | | |
| **GVIF** | | **Df** | | **GVIF^(1/(2*Df))** | **Interacts With** | **Other Predictors** | | | | | |
| 1.2251835 | | 7 | | 1.014612205 | newgroups, week | MVPA_SR_baseline, Age, gender, correctmood_baseline, BMI_baseline, olanzapineequivalents, Durationillness, amotivation_baseline, controlledregulation_baseline | | | | | |
| 1.2251835 | | 7 | | 1.014612205 | timepoint, week | MVPA_SR_baseline, Age, gender, correctmood_baseline, BMI_baseline, olanzapineequivalents, Durationillness, amotivation_baseline, controlledregulation_baseline | | | | | |
| 1.2251835 | | 7 | | 1.014612205 | timepoint, newgroups | MVPA_SR_baseline, Age, gender, correctmood_baseline, BMI_baseline, olanzapineequivalents, Durationillness, amotivation_baseline, controlledregulation_baseline | | | | | |
| 1.3878073 | | 1 | | 1.178052325 | -- | timepoint, newgroups, week, Age, gender, correctmood_baseline, BMI_baseline, olanzapineequivalents, Durationillness, amotivation_baseline, controlledregulation_baseline | | | | | |
| 1.6401837 | | 1 | | 1.280696573 | -- | timepoint, newgroups, week, MVPA_SR_baseline, gender, correctmood_baseline, BMI_baseline, olanzapineequivalents, Durationillness, amotivation_baseline, controlledregulation_baseline | | | | | |
| 1.1995247 | | 1 | | 1.095228171 | -- | timepoint, newgroups, week, MVPA_SR_baseline, Age, correctmood_baseline, BMI_baseline, olanzapineequivalents, Durationillness, amotivation_baseline, controlledregulation_baseline | | | | | |
| 1.5857091 | | 1 | | 1.25924942 | -- | timepoint, newgroups, week, MVPA_SR_baseline, Age, gender, BMI_baseline, olanzapineequivalents, Durationillness, amotivation_baseline, controlledregulation_baseline | | | | | |
| 1.6317201 | | 1 | | 1.277388012 | -- | timepoint, newgroups, week, MVPA_SR_baseline, Age, gender, correctmood_baseline, olanzapineequivalents, Durationillness, amotivation_baseline, controlledregulation_baseline | | | | | |
| 1.2809032 | | 1 | | 1.131769948 | -- | timepoint, newgroups, week, MVPA_SR_baseline, Age, gender, correctmood_baseline, BMI_baseline, Durationillness, amotivation_baseline, controlledregulation_baseline | | | | | |
| 2.3649979 | | 1 | | 1.537854963 | -- | timepoint, newgroups, week, MVPA_SR_baseline, Age, gender, correctmood_baseline, BMI_baseline, olanzapineequivalents, amotivation_baseline, controlledregulation_baseline | | | | | |
| 1.2105067 | | 1 | | 1.100230292 | -- | timepoint, newgroups, week, MVPA_SR_baseline, Age, gender, correctmood_baseline, BMI_baseline, olanzapineequivalents, Durationillness, controlledregulation_baseline | | | | | |
| 1.6974066 | | 1 | | 1.302845567 | -- | timepoint, newgroups, week, MVPA_SR_baseline, Age, gender, correctmood_baseline, BMI_baseline, olanzapineequivalents, Durationillness, amotivation_baseline | | | | | |
| Durationillness - duration of illness  BMI – body mass index  Newgroups – exercise groups  MVPA_SR _baseline– self report moderate to vigorous physical activity  Correctmood- baseline mood  Controlledregulation – controlled regulation  Olanzapine equivalents **Appendix 11.** Univariate analyses. | | | | | | | | | | | |
| FATIGUE | |  | | |  |  |  |  |  |  |  |
| **effect** | | **term** | | | **estimate** | **std.error** | **statistic** | **df** | **p.value** |  |  |
| fixed | | (Intercept) | | | 9.307692 | 0.950594 | 9.791447 | 130.004 | 2.7E-17 |  |  |
| fixed | | timepointpre | | | 1.884615 | 1.041311 | 1.809849 | 141.0425 | 0.072448 |  |  |
| fixed | | newgroups | | | 3.307692 | 1.344343 | 2.460452 | 130.004 | 0.015188 |  |  |
| fixed | | week8 | | | -0.12316 | 1.068839 | -0.11523 | 142.9913 | 0.908423 |  |  |
| fixed | | timepointpre:newgroups | | | -3.07692 | 1.472636 | -2.0894 | 141.0425 | 0.038468 |  |  |
| fixed | | timepointpre:week8 | | | 0.532051 | 1.503003 | 0.353992 | 141.0425 | 0.723873 |  |  |
| fixed | | newgroups:week8 | | | -0.21337 | 1.511567 | -0.14116 | 142.9913 | 0.887944 |  |  |
| fixed | | timepointpre:newgroups:week8 | | | -1.42308 | 2.125567 | -0.6695 | 141.0425 | 0.504268 |  |  |
|  | |  | | |  |  |  |  |  |  |  |
| POSITIVE WELLBEING | | | | |  |  |  |  |  |  |  |
| **effect** | | **term** | | | **estimate** | **std.error** | **statistic** | **df** | **p.value** |  |  |
| fixed | | (Intercept) | | | 20.26923 | 0.837945 | 24.18921 | 91.9203 | 1.24E-41 |  |  |
| fixed | | timepointpre | | | -2.96154 | 0.729635 | -4.05893 | 142.6553 | 8.09E-05 |  |  |
| fixed | | newgroups | | | 0.192308 | 1.185033 | 0.16228 | 91.9203 | 0.871441 |  |  |
| fixed | | week8 | | | 1.246961 | 0.750364 | 1.661807 | 143.8118 | 0.09873 |  |  |
| fixed | | timepointpre:newgroups | | | -0.07692 | 1.03186 | -0.07455 | 142.6553 | 0.940679 |  |  |
| fixed | | timepointpre:week8 | | | -1.03846 | 1.053138 | -0.98606 | 142.6553 | 0.325771 |  |  |
| fixed | | newgroups:week8 | | | -1.55058 | 1.061176 | -1.46119 | 143.8118 | 0.146144 |  |  |
| fixed | | timepointpre:newgroups:week8 | | | 0.576923 | 1.489362 | 0.387363 | 142.6553 | 0.699065 |  |  |
|  | |  | | |  |  |  |  |  |  |  |
| PSYCHOLOGICAL DISTRESS | | | | |  |  |  |  |  |  |  |
| **effect** | | **term** | | | **estimate** | **std.error** | **statistic** | **df** | **p.value** |  |  |
| fixed | | (Intercept) | | | 5.269231 | 0.726138 | 7.256513 | 111.6146 | 5.62E-11 |  |  |
| fixed | | timepointpre | | | 2.423077 | 0.736121 | 3.291683 | 140.0407 | 0.001261 |  |  |
| fixed | | newgroups | | | 1.115385 | 1.026914 | 1.086152 | 111.6146 | 0.279752 |  |  |
| fixed | | week8 | | | 0.9499 | 0.7562 | 1.25615 | 141.7083 | 0.211129 |  |  |
| fixed | | timepointpre:newgroups | | | 0.153846 | 1.041032 | 0.147782 | 140.0407 | 0.882727 |  |  |
| fixed | | timepointpre:week8 | | | 0.785256 | 1.062499 | 0.739065 | 140.0407 | 0.461104 |  |  |
| fixed | | newgroups:week8 | | | -1.03788 | 1.069428 | -0.9705 | 141.7083 | 0.333451 |  |  |
| fixed | | timepointpre:newgroups:week8 | | | -1.11218 | 1.502601 | -0.74017 | 140.0407 | 0.460436 |  |  |
|  | |  | | |  |  |  |  |  |  |  |
